# Detecting neuropsychiatric fluctuations in Parkinson’s Disease using patients’ own words: the potential of large language models

**DOI:** 10.1038/s41531-025-00939-8

**Published:** 2025-04-18

**Authors:** Matilde Castelli, Mario Sousa, Illner Vojtech, Michael Single, Deborah Amstutz, Marie Elise Maradan-Gachet, Andreia D. Magalhães, Ines Debove, Jan Rusz, Pablo Martinez-Martin, Raphael Sznitman, Paul Krack, Tobias Nef

**Affiliations:** 1 ARTORG Center for Biomedical Engineering Research, Gerontechnology and Rehabilitation Group, University of Bern, Bern, Switzerland; 2 Department of Neurology, Bern University Hospital and University of Bern, Bern, Switzerland; 3 Department of Circuit Theory, Faculty of Electrical Engineering, Czech Technical University in Prague, Prague, Czech Republic; 4 Center for Networked Biomedical Research in Neurodegenerative Diseases (CIBERNED), Carlos III Institute of Health, Madrid, Spain; 5 ARTORG Center for Biomedical Engineering Research, AIMI, University of Bern, Bern, Switzerland

**Keywords:** Parkinson's disease, Human behaviour

## Abstract

Over the past decade, neuropsychiatric fluctuations in Parkinson’s disease (PD) have been increasingly recognized for their impact on patients’ quality of life. Speech, a complex function carrying motor, emotional, and cognitive information, offers potential insights into these fluctuations. While previous studies have focused on acoustic analysis to assess motor speech disorders reliably, the potential of linguistic patterns associated with neuropsychiatric fluctuations in PD remains unexplored. This study analyzed the content of spontaneous speech from 33 PD patients in ON and OFF medication states, using machine learning and large language models (LLMs) to predict medication states and a neuropsychiatric state score. The top-performing model, the LLM Gemma-2 (9B), achieved 98% accuracy in differentiating ON and OFF states and its predicted scores were highly correlated with actual scores (Spearman’s ρ = 0.81). These methods could provide a more comprehensive assessment of PD treatment effects, allowing remote neuropsychiatric symptom monitoring via mobile devices.

## Introduction

Parkinson’s disease (PD) is a highly heterogeneous neurodegenerative disorder that presents with diverse symptoms, underlying causes, and individual patient experiences^1^. While historically, PD research has predominantly focused on its motor symptoms, it has become increasingly evident that PD is not just a motor disorder but also a complex neuropsychiatric condition^2^. Neuropsychiatric symptoms, such as mood changes, depression, anxiety, apathy, impulse control disorders, and cognitive impairment, have an equal or even greater impact on patients’ quality of life than motor symptoms^3^.

Dopaminergic replacement therapies are effective not only in alleviating the motor symptoms of PD but also play a critical role in managing common neuropsychiatric symptoms.

Nonetheless, as the disease progresses, the therapeutic window of optimal symptom control narrows. Consequently, the long-term use of dopaminergic medication and disease progression often lead to fluctuations in symptoms, which in the case of neuropsychiatric symptoms are commonly called neuropsychiatric fluctuations^4^. During “OFF” medication states (low levels of dopaminergic medication), patients frequently report fatigue, low mood, lack of initiative, and difficulty generating new ideas. Conversely, during “ON” medication states (high levels of dopaminergic medication), patients may exhibit increased talkativeness, euphoria, impulsivity, and rapid generation of new ideas^5,6^. Recognizing these fluctuations is essential since they can substantially impact patients’ daily functioning and overall well-being^4^.

Timely adjustments to treatment can prevent the emergence of more severe behavioral issues, such as apathy or impulse control disorders, which can profoundly impact both the patient’s and their family’s lives^6–8^. Therefore, at this stage of PD, the patient’s quality of life heavily relies on the clinician’s ability to optimally adjust dopaminergic treatment, minimizing “OFF” periods and mitigating medication peaks^9^. However, the precise management of these therapies is often challenging due to the limited and infrequent assessments in clinical practice. Additionally, patients usually find it difficult to retrospectively report past neuropsychiatric fluctuations, as well as their previous mood and behavior states.

Speech analysis offers a promising solution to overcome the challenges of monitoring PD symptoms^10^. Previous studies have demonstrated that paralinguistic speech features (e.g., prosodic, respiratory, phonatory, and averaged speech spectral features) are valuable digital biomarkers for assessing motor severity, monitoring disease progression, and tracking changes in motor symptoms due to dopaminergic effects^11–14^. Speech can also provide rich insights into cognition and emotions, with prosodic features being the most studied paralinguistic domain for emotion recognition^15–18^. However, prosodic features in PD are heavily influenced by core motor symptoms, and the resulting dysarthria may alter emotional perception, often skewing it towards negative emotions, such as sadness^19^.

Recent advances in natural language processing have facilitated the exploration of language patterns in PD, revealing linguistic abnormalities in the language of patients with PD compared to healthy individuals^20–22^. Most existing studies have primarily evaluated linguistic features extracted from spontaneous discourse as markers of cognitive functions^23,24^. However, language conveys a broader range of valuable information, including feelings and emotions^25,26^. By analyzing “what patients say”, we can move beyond standardized patient-reported outcomes, gaining deeper insights into patients’ individual experiences and identifying the most bothersome symptoms in PD^26^.

In this study, we used natural language processing techniques to assess neuropsychiatric changes induced by dopaminergic medications, integrating recent advances in generative artificial intelligence (AI) and large language models (LLMs). First, we analyzed the semantic meaning conveyed by the free speech samples to discriminate between “ON” and “OFF” medication states using traditional machine learning (ML) algorithms and recent LLMs. Then, we evaluated the ability of these models to predict a neuropsychiatric state score under the two medication states.

## Results

### Participants

This study involved 35 patients with PD who completed the speech assessment during the levodopa challenge and provided written consent for biomedical research. One patient was excluded due to a revised diagnosis after the levodopa assessment. After revising speech recordings, another patient was excluded as their answers consisted solely of single words.

The participants’ mean age was 64 ± 7.7 years, and disease duration were 10.3 ± 3.4 years. Their detailed demographic and clinical information are provided in Table 1.

**Table 1:** Demographic and clinical characteristics of study participants

Patients with PD	*N* = 33
Demographic characteristics	
Sex assigned at birth (M/F)	24/9
Age (years), mean ± STD (min-max)	64.3 ± 7.7 (48–77)
Languages*, n* (%)	
- Swiss-German	21 (63.7%)
- French	8 (24.2%)
- Italian	4 (12.1%)
Disease duration (years) ± STD, (min-max)	10.3 ± 3.4 (3.0–18.0)
Clinical characteristics, mean ± STD (min-max)	
MDS-UPDRS I	12.8 ± 5.8 (2.0–28.0)
MDS-UPDRS II	15.5 ± 6.8 (2.0–26.0)
MDS-UPDRS III: OFF medication	44.1 ± 12.0 (24.0–73.0)
MDS-UPDRS III: ON medication	19.7 ± 7.3 (7.0–38.0)
MDS-UPDRS IV	10.5 ± 3.9 (0.0–17.0)
Neuropsychiatric State Score: ON medication	48.2 ± 8.7 (26.0–60.0)
Neuropsychiatric State Score: OFF medication	17.2 ± 12.0 (0.0–49.0)
QUIP-RS	9.9 ± 11.3 (0.0–41.0)
SAS	10.5 ± 4.2 (3.0–19.0)
HADS -Anxiety	5.9 ± 3.9 (0.0–15.0)
HADS-Depression	4.2 ± 3.0 (0.0–16.0)
MoCA	25.9 ± 2.7 (20–30)
Average Speech Length (seconds)	44.8 ± 14.9 (15.2–106)
Speech length in ON medication	43.7 ± 11.8 (15.2-76.7)
Speech length in OFF medication	45.9 ± 17.6 (17.1-106)

*PD* Parkinson’s disease, *F* female, *M* male, *STD* standard deviation, *MDS-UPDRS* Movement Disorder Society–Unified Parkinson’s Disease Rating Scale, *QUIP-RS* Questionnaire for Impulsive-Compulsive Disorders in Parkinson’s Disease Rating Scale, *HADS* Hospital Anxiety and Depression Scale, *MoCA* Montreal Cognitive Assessment.

**Fig. 1:**
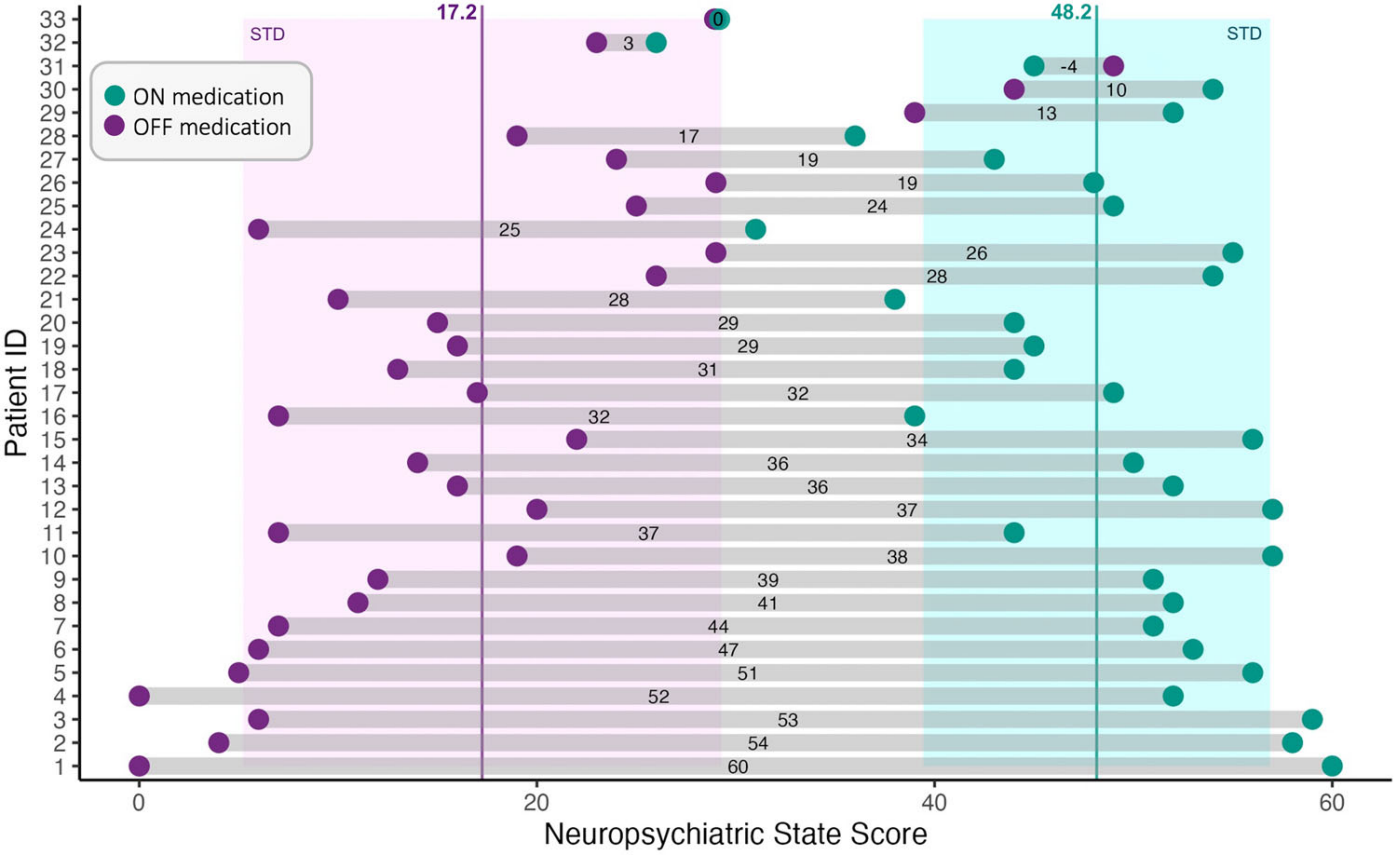
Neuropsychiatric State Score during OFF and ON medication states. The dots represent the neuropsychiatric state score for each participant during the OFF-medication condition (purple dots) and ON-medication condition (green dots). The values on the gray segments indicate the difference between the scores in the two states. The vertical lines and the shaded areas represent our sample’s average neuropsychiatric state score and the standard deviation in the two conditions. ID identifier, STD standard deviation.

Figure 1 shows the variability in the neuropsychiatric scores between the ON and OFF medication states, with a mean difference of 31.2 ± 14.9 out of a maximum difference of 60. The average speech length was comparable between the ON and OFF medication states (ON 43. ± 11.8 vs. OFF 45.9 s ± 17.6, W = 263.0, *p* = 0.76, Wilcoxon signed-rank test).

### ON-OFF medication state classification

**Fig. 2:**
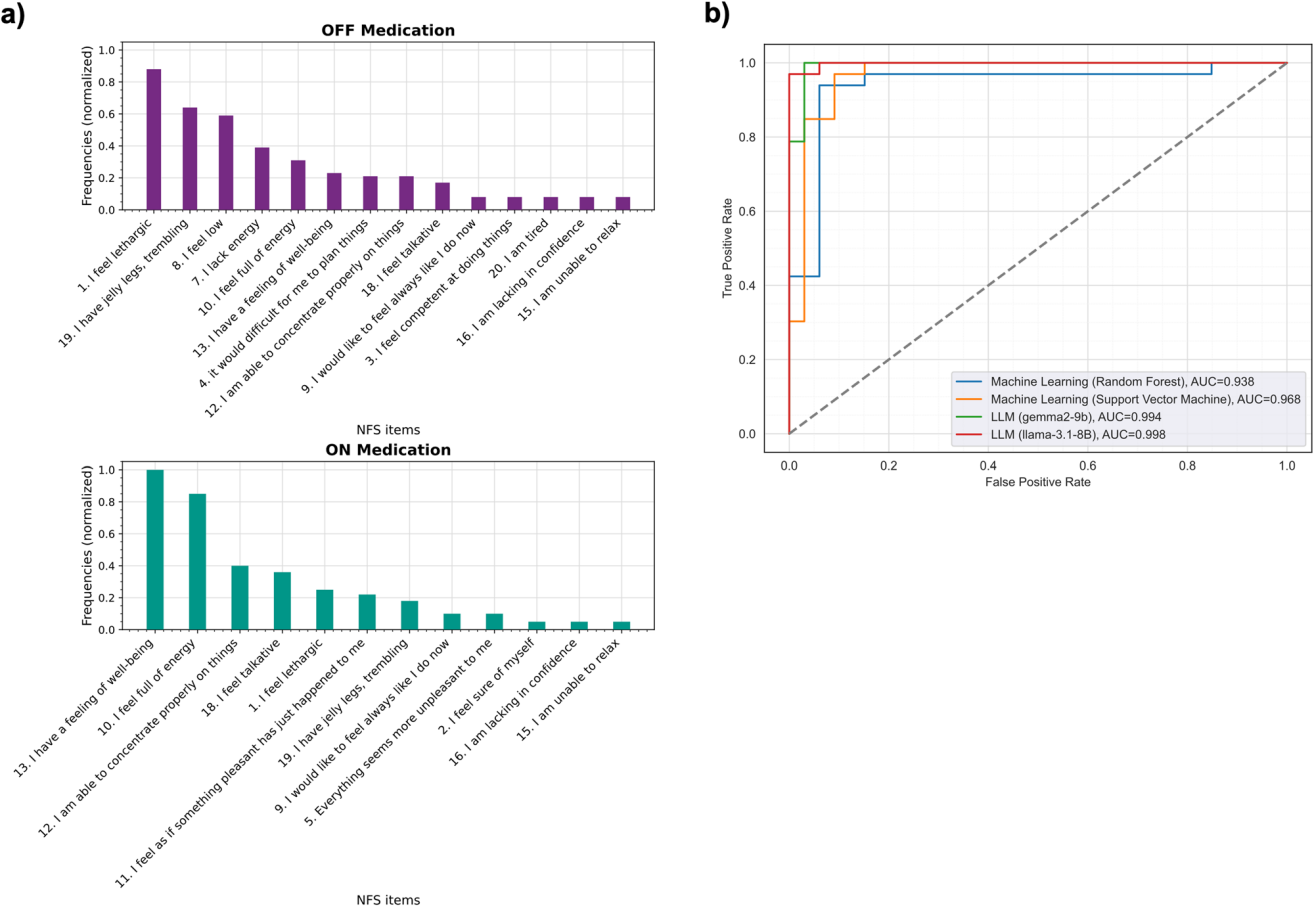
Additional metrics for best models in each approach. **a** Illustrates the frequencies of the top five most similar items of the Neuropsychiatric Fluctuation Scale (NFS) retrieved using the semantic search approach. The x-axis shows the 20 NFS items while the y-axis shows their normalized frequencies, indicating how frequently each item was selected as one of the top five most similar to the recordings. This approach predominantly assigned OFF items as most similar to the language in the OFF-medication state and ON items to recordings in the ON-medication state. **b** Shows the receiver operating characteristic (ROC) and the corresponding area under the curve (AUC) values obtained with the top performing machine learning models and the LLMs.

Table 2 presents the best results obtained with each method. The semantic search approach achieved a classification accuracy of 0.85 when the “*multi-qa-mpnet-base-dot-v1*” model was used to compute the text embeddings, and the top five most similar NFS items were used as voters. Figure 2a shows the occurrences of the five most frequent similar NFS items retrieved by this method.

**Table 2:** Comparison of the top-performing models for each approach in classifying medication state

Medication State Classification Approach	Model	Accuracy	Precision	Recall	F1 macro
Semantic Search	multi-qa-mpnet-base-dot-v1	0.848	0.847	0.848	0.848
Machine Learning	gte-Qwen2-1.5B-instruct. + Random Forests, gte-Qwen2-1.5B-instruct. + Support Vector Machine	0.924	0.925	0.924	0.924
LLMs	Gemma-2 (9B), Llama-3.1 (8B) (few-shot setting)	**0.984**	**0.984**	**0.984**	**0.984**

Best performances are highlighted in bold.

In the second approach, we used three ML classifiers (RF, SVM, and Gaussian NB) to classify the speech transcriptions. An accuracy and F1-score of 0.92 were obtained when the RF and the SVM classifiers were applied to the “*Alibaba-NLP/gte-Qwen2-1.5B-instruct*” text embeddings.

In the third approach, we instructed three different LLMs to classify the transcriptions directly. The LLMs Gemma-2 (9B) and Llama-3.1 (8B) achieved an accuracy of 0.98 in the few-shot setting. Specifically, the Gemma-2 (9B) and Llama-3.1 (8B) achieved the best results when the six and ten most similar recordings to the query were used in the prompt, respectively (Supplementary Fig. 2).

The best models were further evaluated by plotting the ROC curves and computing the area under the ROC curve (AUC) (Fig. 2b). The LLMs (Gemma-2 (9B) and Llama-3.1 (8B)) achieved an AUC of 0.99, followed by the machine learning methods, which achieved an AUC of 0.94 with RF and 0.97 with SVM.

The LLMs (Gemma-2 (9B) and Llama-3.1 (8B)) misclassified only one recording. For example, the Gemma-2 (9B) misclassified one sample belonging to the OFF-medication class, assigning the label “ON” with a probability of 0.73. This recording belonged to patient 31 (Fig. 1), who had a neuropsychiatric state score of 49 in the OFF-medication state versus 45 in the ON-medication state. This patient was the only one with a higher ON-score than the OFF-score and was among three patients with a fluctuation of < 10/60, indicating the absence of clinically meaningful neuropsychiatric fluctuations.

### Neuropsychiatric state score predictions

We used similar methods to predict the neuropsychiatric state score. The top-performing models for each approach are shown in Table 3. The semantic search approach obtained an RMSE of 14.0 when the top five most similar patients’ recordings were considered. The RF model receiving the “*Alibaba-NLP/gte-Qwen2-1.5B-instruct”* embeddings as input achieved an RMSE of 11.2, MAE of 8.68, and a median absolute error of 6.23.

**Table 3:** Comparison of the top-performing models across the three approaches for predicting the neuropsychiatric state score

Neuropsychiatric State Score Prediction Approach	Model	RMSE	MAE ± STD (Median AE, Q1-Q3)	R^2^
Semantic search	multi-qa-mpnet-base-dot-v1	14.0	11.7 **±** 7.9 (11.0, 5.3–17.0)	0.43
Machine learning	gte-Qwen2-1.5B-instruct + Random Forests	11.2	8.7 ± 7.1 (6.2, 3.1–13.4)	0.64
LLM	Gemma-2 (9B) (few-shot setting)	**10.6**	**8.1** ± **6.9 (6.0, 3.0–10.4)**	**0.68**

*MAE* mean absolute error, *AE* absolute error, *Q1* first quartile, *Q3* third quartile, *RMSE* root mean squared error, *STD* standard deviation, *LLM* Large Language Model.

Best results are highlighted in bold.

**Fig. 3:**
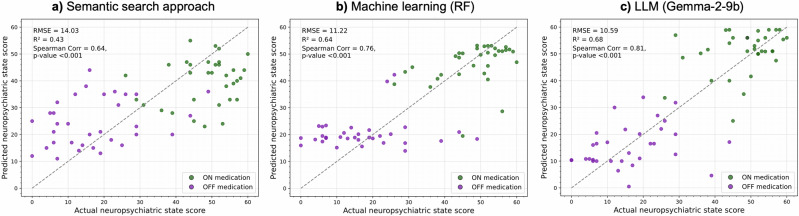
Scatter plots of predicted vs. actual neuropsychiatric state score. The gray dotted line represents perfect correlation. **a** Shows the results of the semantic search approach based on the similarities between speech transcriptions. **b** Reports the score estimates of the machine learning model (Random Forest) applied to gte-Qwen2-1.5B-instruct embeddings. **c** Illustrates the predictions computed by Gemma 2-9b. The colored dots represent the average estimates of 9 predictions. RMSE Root Mean Squared Error, R^2^ coefficient of determination.

The LLM “gemma-2-9b” in the nine-shot setting resulted in an RMSE of 10.66 and MAE of 8.1. Figure 3 shows scatter plots showcasing the relationships between predicted and actual neuropsychiatric state scores with the best-performing models for each approach. The LLM Gemma-2 (9B) predictions showed the strongest correlation with the actual neuropsychiatric state scores (Spearman’s *ρ* = 0.81, *p* < 0.001; Fig. 3c), followed by the Random Forest model (*ρ* = 0.76, *p* < 0.001; Fig. 3b) and the semantic search (*ρ* = 0.64, *p* < 0.001; Fig. 3a).

The RF model predicted similar scores for the transcriptions recorded in the OFF-medication state, assigning values between 15 and 25. All the approaches better estimated the neuropsychiatric scores in the ON-medication state. The MAEs were 6.6, 7.5, and 11.1 in the ON-medication state and 10.7, 8.6, and 12.2 in the OFF-medication state for the ML model (RF), Gemma-2 (9B) LLM and semantic search, respectively.

## Discussion

This study explored multiple linguistic approaches to predict medication states in patients with PD using a spontaneous free speech paradigm. The LLMs Gemma-2 (9B) and Llama-3.1 (8B) achieved the highest accuracy (0.98) and AUC (0.99) for classifying the medication state in the few-shot setting. The other approaches also performed strongly, with text embedding combined with RF and SVM achieving an accuracy of 0.92 and an AUC of 0.94 and 0.97, respectively.

The medication states were classified based on the assumption that most patients with PD experience improved neuropsychiatric symptoms in the “ON” medication state (indicated by higher neuropsychiatric state scores) and worsening symptoms in the OFF medication state (indicated by lower neuropsychiatric state scores). However, given the heterogeneity of PD, it is unsurprising that in rare cases, patients may not exhibit sharp neuropsychiatric fluctuations across medication states or may even experience worsening neuropsychiatric symptoms during the ON medication state. These scenarios may lead to model misclassifications but reflect the actual symptom patterns of the patients. For example, the Gemma-2 (9B) LLM misclassified one patient with consistently high neuropsychiatric state scores (neuropsychiatric state score >40) in both medication states, as this patient showed minimal neuropsychiatric changes between states. These cases highlight variability in PD symptomatology but demonstrate that Gemma-2 (9B) LLM performed robustly even in complex situations.

Performance metrics alone did not clarify whether the models based their predictions on the neuropsychiatric content of patients’ transcriptions or other symptom domains. One possibility to enhance the transparency of the black-box LLMs is to generate self-explanations along with their predictions. Therefore, we “asked” the LLMs to explain their predictions, including the most discriminative words for the medication states. The most frequent words or phrases self-retrieved by the model were “feel better” and “relaxed” for the ON medication state and “don’t feel well” and “tired” for the OFF medication state. While these terms primarily relate to neuropsychiatric symptoms, in some cases, the generated self-explanations just reflected the prompts we provided. This finding is consistent with previous research, which showed that although plausible, the faithfulness of generated explanations can be unreliable^27^. This issue is particularly evident when using few-shot prompting, where the LLM models tend to mimic the provided human explanation rather than mirroring their inner decision-making^28^.

Moreover, in Supplementary Material (Section F), we included a calibration analysis of the best-performing models, which were not well calibrated. While our primary goal was to demonstrate the potential of using NLP techniques and generative AI for detecting neuropsychiatric fluctuations, calibration remains an important factor to consider when transferring these techniques in clinical practice. As an outlook for future studies, post-processing methods such as temperature scaling^29^ for LLMs could be explored to improve calibration and, thus, confidence reliability.

These caveats underscore the importance of using complementary approaches. For example, while the semantic search approach achieved a slightly lower accuracy of 0.88, it provided additional value by retrieving NFS items closely related to each transcription. Interestingly, the most frequent NFS items used to classify ON states (Fig. 2a) —“I have a feeling of well-being” and “I feel talkative, I want to communicate”—and OFF states—“I have jelly legs, trembling,” “I lack energy for everyday activities,” and “I feel low”— aligned with the most sensitive items for distinguishing medication states in the original NFS validation study^30^. This observation suggests that the items retrieved by the semantic search approach partially mirrored those most effective in differentiating medication states in the NFS validation. Additionally, while the item “I feel lethargic” was frequently noted in both medication states, it occurred more frequently in the OFF state.

Only a few studies have focused on medication state classification. For example, Norel et al.^31^ recently combined linguistic (semantic word embeddings) and acoustic features from three speech tasks to differentiate between ON and OFF medication states. The greatest accuracy (0.89) was achieved with a picture description task, where PD patients were observed to use fewer action-related words in the OFF state, consistent with previous research. However, their work could not disentangle whether the medication prediction was driven by changes in motor, emotional, or cognitive symptom domains since it lacked a measure for neuropsychiatric fluctuations. Similarly, our study could not disentangle emotional and cognitive symptom domains, as both are represented in the NFS. However, using AI to analyze semantics seems to provide a good measure of the cognitive-emotional state while traditional acoustic analysis instead focuses on motor states. Based on our new findings, speech content analysis appears to provide an even superior prediction of medication states than the traditional speech acoustic analysis^10^. Combining both approaches with the same speech recordings might be of value, considering that acoustic analysis does not rely on a black box with potential sources of bias, unlike LLMs.

Restricting the classification of the neuropsychiatric state in patients with PD to only ON and OFF medication states may overlook intermediate states, as some patients may not experience a sharp neuropsychiatric transition between them. To account for this continuum, we evaluated the accuracy of the different models in predicting the neuropsychiatric state scores. Consistent with the medication classification results, the LLMs outperformed the other approaches, particularly Gemma-2 (9B), which achieved the lowest RMSE of 10.6 and MAE of 8.1, showing a strong correlation (*ρ* = 0.81) between predicted and actual neuropsychiatric state scores. Promising accuracies in neuropsychiatric state score prediction were also achieved using RF (RMSE = 11.22), outperforming the LLMs in the zero-shot scenario and the other traditional ML models. Moreover, it achieved the lowest MAE in estimating the recordings in the ON medication state.

The LLMs performed best when a few of the most semantically similar transcriptions were included as input (few-shot setting). A significant advantage of LLMs is their ability to adapt to new tasks by “learning” from a few relevant examples^32^. This is extremely valuable in the medical field, where small sample sizes are common and pose a challenge for traditional ML algorithms. Additionally, we used nine paraphrases of the input with the same in-context examples for each transcription to achieve stable and consistent predictions across different prompts^33^. Indeed, LLMs have the drawback of being extremely sensitive to the prompt’s format and content^34^. Varying the prompts, especially changing in-context examples, resulted in different classification outcomes and neuropsychiatric state score predictions. We observed that incorporating semantically similar transcriptions into the prompt, instead of including random examples, reduced the prediction errors and drastically decreased the variance in the repeated estimates (Supplementary Fig. 5). Moreover, throughout the prompt tuning process, various issues with LLM-generated outputs occurred, including inconsistent output formats (particularly in the zero-shot setting), the generation of multiple labels instead of a single one, and unstructured text arising from a reluctance to classify the neuropsychiatric state of a person. These issues were overcome using the reported final version of the prompt. Such findings underscore the importance of carefully engineering prompts and selecting in-context examples when utilizing LLMs for neuropsychiatric assessment in patients with PD. By optimizing these parameters, we improved the predictive performance and reliability of the models, further validating the use of LLMs to capture neuropsychiatric fluctuations associated with dopaminergic medication states.

Despite our promising results, our study had several limitations. Firstly, its sample size was relatively small for ML applications, and the cohort comprised patients with PD with moderate to severe fluctuations, to whom a suprathreshold dose of medication was administered, which could make neuropsychiatric fluctuations more pronounced. Secondly, patients were assessed in a fixed sequence: initially after overnight withdrawal of medication and subsequently after administration of a suprathreshold dose of dopaminergic medication. Although we recognize the possibility of a repetition effect due to increased familiarity with the task in the second assessment, we believe its impact is likely minimal. This assumption is supported by data presented in Supplementary Material (Section G), where repeating the experiments twice in the ‘off’ state for 20% of the patients did not yield significant differences between the two trials conducted under the same conditions. Moreover, all the included patients also underwent comprehensive clinical assessments, which allowed our models to train on high-quality, comprehensive patient data with significant variability between medication states. Nevertheless, our findings must be replicated in larger populations to validate our methods further.

Thirdly, our approach to estimating neuropsychiatric fluctuations was based on the NFS scale, which has shown reliability in quantifying acute neuropsychiatric fluctuations^30,35^. However, large-scale validation of the neuropsychiatric state score has not yet been reported (ClinicalTrials.gov: NCT04455074), limiting the generalizability of our findings.

Fourthly, our study relies on Whisper-generated automatic transcriptions, which can sometimes produce hallucinations. While Whisper performs well, even with Swiss German dialects^36,37^, concerns remain about its reliability in high-risk settings, with around 1% of transcriptions affected by hallucinations^38^. In our study, occasional hallucinations (around 2 out of 66 sentences were affected), specifically as repeated phrases at the end of transcriptions. These were easily identifiable and did not alter semantic meaning. However, a thorough quantitative evaluation, especially for speech from patients with Parkinson’s disease, would be important to detect other potential hallucinations at lexical and semantic levels.

Fifthly, these methods were based on the assumption that the speech contained emotion-enriched content. Notably, the models predicted neuropsychiatric state scores more accurately for ON recordings. This observation could be explained by the fact that, in the OFF state, some patients tended to focus more on motor symptoms and provide limited information about their neuropsychiatric condition, potentially affecting the neuropsychiatric state score predictions. While this could be considered a limitation, it also underscores the robustness of our methods in isolating neuropsychiatric features from other symptom domains.

Sixthly, while translating the transcriptions into English allows the application of these methods across different languages, it could introduce some additional biases and not fully capture the nuances of the cultures or expressions^39^. For example, the NFS item “At the moment, I have jelly legs, trembling” may carry a predominantly neuropsychiatric connotation in French but can be interpreted as a motor symptom in German. Future work could explore using original transcriptions and multilingual LLMs to better capture these cultural nuances of different languages.

Additionally, both LLMs (in the few-shot setting) and ML models made their predictions based on examples from other patients, which vary in their characteristics. Collecting repeated recordings from the same patient could lead to the development of more personalized, patient-specific methods, potentially improving long-term monitoring.

Finally, we selected relatively small LLMs due to limited computational power. Models with a larger size should be considered to further improve the predictions, leveraging techniques such as quantization to reduce computational and memory requirements^40,41^.

By analyzing patients’ own words describing their condition, our methods enhance the sensitivity and specificity of symptom detection and provide a foundation for developing real-time monitoring systems of neuropsychiatric fluctuations. Based on brief talks, our approach may provide a more natural and engaging method for collecting neuropsychiatric self-reported outcomes than traditional tools. Indeed, conventional clinical instruments used in routine clinical practice and research settings rely on pre-defined, structured responses, which can limit patients’ ability to fully express their experiences and potentially lead to inaccurate symptom reporting (e.g., underreporting). These tools are also impractical for frequent daily use, making them less suitable for capturing the fluctuating symptoms of PD outside clinical settings. In contrast, smartphone-based speech assessments, based, for example, on daily calls^42^, offer the potential to remotely and unobtrusively monitor PD progression^10^. Given the minimal effort required from patients, our approach could be easily adapted for home use by developing smartphone applications, enabling continuous, unobtrusive, remote monitoring of patients’ neuropsychiatric symptoms^10^. Moreover, our methods based on speech content analysis provide the additional advantage over acoustic analysis of being less sensitive to the quality of smartphone microphones^10^.

In conclusion, our proof-of-concept study demonstrates the feasibility of using the patients’ speech content to accurately differentiate between medication states and predict neuropsychiatric fluctuations. This approach can lead to more timely and personalized adjustments of therapeutic strategies, allowing for the detection of neuropsychiatric fluctuations. These fluctuations significantly impact patients’ quality of life directly^4^ and underlie behavioral sensitization, driving potentially devastating behavioral complications of dopaminergic treatment, such as impulse control disorders, punding, and dopamine dysregulation syndrome^8,43^.

## Methods

### Study design

This observational study included patients diagnosed with fluctuating PD according to Movement Disorders Society criteria^44^. These patients underwent a levodopa challenge as part of the routine evaluation for advanced PD therapies, which included systematic recording of spontaneous speech in their native languages. Patients with PD clinically diagnosed with dementia, according to the Diagnostic and Statistical Mental of Mental Disorders, Fifth Edition^45^, were excluded. The patients were recruited between 2021 and 2023 at the University Hospital Inselspital in Bern, Switzerland.

The participants were evaluated in ON and OFF medication states. Initially, they were assessed after an overnight OFF, practically defined as a withdrawal of at least eight hours for levodopa and/or 48 h for dopaminergic agonists. Then, they were reevaluated in the ON medication state, occurring 30–60 min after taking a fast-acting compound of levodopa/benserazide 100/25 mg, equivalent to 150% of the patient’s usual dopaminergic equivalent morning dose. Levodopa equivalent doses were calculated according to previously described conversion factors^46^. Additionally, to ensure that the results reported were not due to a learning effect and more familiarity with the task and the interviewer, a subgroup of 7 PD patients (about 20% of the total sample) was assessed three times. Two times in the practically OFF medication condition with an interval of 15 min, and one time in the practically defined ON condition.

The retrospective analysis of the data collected from patients who provided general consent for biomedical research was approved by the local ethics committee (KEK 2023-01427) and conducted according to the Declaration of Helsinki.

### Clinical examination

The neuropsychological fluctuations were quantified using the Neuropsychiatric Fluctuations Scale (NFS)^47^ in both ON and OFF medication states. The NFS is a self-report questionnaire designed to assess the momentary neuropsychiatric symptoms of PD, demonstrating good sensitivity in detecting acute changes in neuropsychiatric symptoms^35,47^. It consists of 20 items rated on a scale from 0 (does not describe how I feel right now) to 3 (describes a lot of how I feel right now), with 10 items reflecting typical ON symptoms (e.g., increased well-being, self-confidence) and the other 10 corresponding to OFF symptoms (e.g., tiredness, anxiety). A neuropsychiatric state score was calculated from the NFS items using a formula previously described by Magalhães et al.^35^, ranging between 0 and 60. A score of 0 indicates a very low mood, whereas a score of 60 corresponds to a very high mood.

Motor symptom severity was assessed in both ON and OFF medication states by the same trained clinician using the Movement Disorder Society–Unified Parkinson’s Disease (MDS-UPDRS) Part III^48^. Non-motor symptoms (MDS-UPDRS Part I), activities of daily living (Part II), and severity of motor complications in daily life (Part IV) were evaluated exclusively in the ON condition.

Global cognitive functioning was assessed using the Montreal Cognitive Assessment (MoCA)^49^.

Depressive and anxiety symptoms were measured using the Hospital Anxiety and Depression Scale (HADS)^50^, apathy was assessed using the Starkstein Apathy Scale (SAS)^51^, and impulse control behavioral disorders were evaluated using the Questionnaire for Impulsive-Compulsive Disorders in Parkinson’s Disease Rating Scale (QUIP-RS)^52^. These assessments, which evaluate chronic cognitive and neuropsychiatric symptoms, were conducted only once during the ON medication state.

### Speech examination

Participants’ speech was recorded in a quiet room using a head-mounted condenser microphone (Shure Beta 53; Shure, Niles, IL, USA) positioned 5 cm from the mouth. The recordings were sampled at 48 kHz with 16-bit resolution^53^. Speech recording was conducted prior to the motor examination to minimize potential biases in participants’ descriptions toward motor symptoms.

Participants were asked to speak freely in a monologue task designed to assess their neuropsychological state under different medication states. To elicit responses rich in emotional and cognitive content, participants were informed of the importance of describing not only their current motor state but also their emotional and cognitive state in detail. The task began with the same open-ended question: “How do you feel right now?”. To replicate a conversational setting, the examiner, who was alone in the room, was facing the participant and maintained an encouraging demeanor. If the initial response was too short, after a pause of 10 s, the examiner would prompt further elaboration to ensure the collection of additional speech. The target cumulative duration for the monologue was 60 s (i.e., upper limit) of total recorded speech length, including pauses and hesitations (not accounting for net connected speech time). This task was conducted under both ON and OFF medication states.

### Speech transcription and English translation

The recorded speech samples were transcribed into text using an automatic speech recognition (ASR) tool developed by OpenAI, USA (Whisper)^54^. This ASR tool was selected based on a comparative performance evaluation of different state-of-the-art speech-to-text models^55^. The recorded speech samples included three languages spoken in Switzerland: French, Italian, and German or their respective Swiss dialects. Since a standardized written form does not exist for the Swiss-German dialects, ASR for Swiss-German usually transcribes it into standard High-German text. The selected ASR has shown impressive zero-shot performance for Swiss-German speech^36^, further improved by fine-tuning techniques^37^. Therefore, to transcribe the Swiss-German recordings, we fine-tuned (https://huggingface.co/blog/fine-tune-whisper) the ASR tool (Whisper-medium version) on a publicly available annotated Swiss-German speech dataset (STT4SG-350)^56^. French and Italian recordings were transcribed using the generic version of the ASR tool (Whisper-large-v3 version (https://github.com/openai/whisper)). Then, all the transcriptions were translated from their original languages into English using Whisper’s translation task since multilingual autoregressive language models usually demonstrate superior performance in English^57^.

Additionally, paralinguistic elements of speech, such as filled pauses (e.g., “ehm”), were removed as they do not carry any semantic value. Furthermore, we removed all the references to medication intake or condition in the transcriptions. Utterances spoken by the examiner (i.e., prompts for elaboration following a 10 s pause) were removed from the transcriptions.

### ON/OFF medication state classification using semantic search

**Fig. 4:**
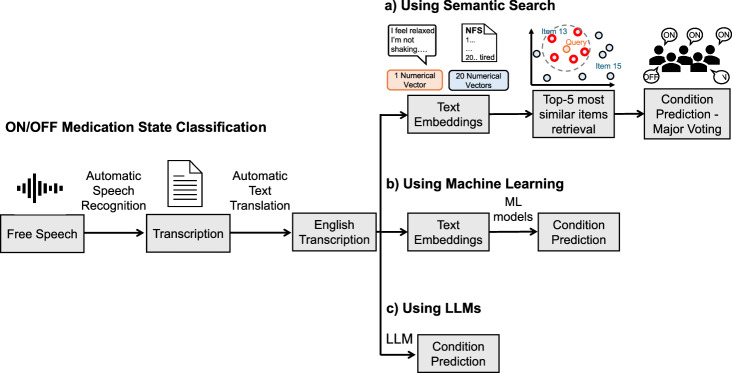
Schematic representation of the three approaches for the ON/OFF medication state classification of speech recordings. The English transcription of each speech recording was the input for all the approaches. **a** The transcriptions (query) and the 20 items (corpus) of the Neuropsychiatric Fluctuation Scale (NFS) are first mapped into numerical vectors. Each recording transcription is treated as a query, and the five most similar NFS items are retrieved using cosine similarity. The query is then classified based on the most frequent label (ON or OFF) among the retrieved items. **b** The medication state is predicted by applying machine learning (ML) models on linguistic features represented as text embeddings. **c** English transcriptions are directly included in a decoder-only Large Language Model (LLM) prompt.

Three approaches were used to classify the medication state from speech recordings. In Fig. 4a, the semantic search approach is presented using the patients’ transcriptions and the NFS items.

A key strength of the NFS questionnaire is that its items were derived from various psychiatric questionnaires completed by a large cohort of PD patients with fluctuations, reflecting the most common neuropsychiatric symptoms of PD^47^. We used this strength to classify participants’ speech based on similarity with the NFS items. First, we performed text normalization; specifically, the speech transcriptions and NFS items were converted to lowercase, punctuation, and specific stop-words (pronouns, conjunctions, articles, and prepositions) were removed while negations were kept. Next, the normalized transcriptions and NFS items were converted into numerical vectors (i.e., text embeddings). Then, the English texts were mapped to a numerical representation using a Bag of n-grams (*n* = 1,2,3) model, implemented with the class *CountVectorizer* from the Python’s *scikit-learn* module (version 1.3.0)^58^. The CountVectorizer, together with the TfidfVectorizer were used as baseline methods. Next, more recent Language Models from Python’s “*sentence-transformer*” module^59^ and an open-source repository (HuggingFace Hub (https://huggingface.co/docs/hub/index)) were used to capture the meaning of the transcriptions. Then, the “*multi-qa-mpnet-base-dot-v1*” model from “*sentence-transformer”*, the “dunzhang/stella_en_1.5B_v5” and the “and “*Alibaba-NLP/gte-Qwen2-1.5B-instruct*” models from the HuggingFace Hub were used to map the text into dense vectors of size 768, 1024, and 1536 respectively. These embedding models were selected based on a tradeoff between GPU memory requirement and their semantic search/retrieval task performance (as reported on the HuggingFace leaderboard (https://huggingface.co/spaces/mteb/leaderboard) and in the “*sentence-transformer*” documentation (https://sbert.net/docs/sentence_transformer/pretrained_models.html).

Each transcription was used as a query, and the cosine similarity was calculated to measure its similarity to each NFS item embedding. For classification, we adopted a simple major voting rule, requiring an odd number of voters to avoid ties. In this approach, the voters corresponded to the NFS items most similar to the query. The optimal number of voters was determined empirically based on classification accuracy (Supplementary Fig. 1). The query was then classified as OFF or ON depending on whether most of the top five most similar NFS items were OFF or ON items. All the results obtained using different text embeddings are reported in Supplementary Table 1.

### ON/OFF medication state classification using machine learning models

First, the speech recordings were mapped into text embeddings as described for semantic search. Given the high dimensionality of the numerical vectors generated from these models (minimum dimension of 768) and the limited data available, we used principal component Analysis (PCA) to the standardized embeddings and Truncated singular value decomposition for the CountVectorizer to reduce their dimensionality. By setting an explained variance threshold of 0.9, the dimensions of the vectors were reduced to 40 for the “*multi-qa-mpnet-base-dot-v1” and for the “dunzhang/stella_en_1.5B_v5”* and 45 for the “*Alibaba-NLP/gte-Qwen2-1.5B-instruct*” embeddings, respectively.

Binary Classification was performed by applying different ML models to the computed text embeddings, such as naïve Bayes (NB), support vector machines (SVM), and Random Forest (RF). The models’ hyperparameters were optimized using the Grid Search approach.

A comprehensive overview of the results obtained using the different text embeddings and machine learning models is reported in Supplementary Table 2.

This approach is graphically represented in Fig. 4b. This ML approach is similar to methods already explored in psychology and mental health to predict depression or dementia from Tweets or speech^60,61^.

### ON/OFF medication state classification using LLMs

Finally, we explored the performance of decoder-only models in classifying different medication states (ON vs. OFF) directly using the transcriptions as input. Different prompts were tested before obtaining consistent and coherent results (see supplementary material section B). In order to instruct the LLMs, the ON and OFF conditions were explained using the NFS items. The final version of the prompt was the following: “*You will be provided with transcription of speech from people with Parkinson’s Disease. Please assign the class ON or OFF to each text. Patients in the OFF condition may experience tiredness, lack of energy, inability to relax, trembling legs, and feel lethargic and low. In the ON condition, they usually feel full of energy, sure of themselves, talkative, and a sense of well-being. Please provide only the class estimate without any additional text*.”

We selected different open-source LLMs based on a tradeoff between benchmark performance according to the HuggingFace Leaderboard and the computational power needed since all the computations were performed locally to ensure the privacy of sensitive data. The selected models are listed in Table 4. We tested these models in both zero-shot (i.e., prompts without any example transcripts included) and few-shot settings (i.e., prompts including some representative transcripts). In the few-shot prompting, in addition to the recording to classify, we included its most semantically similar transcriptions from the other patients, selected using the cosine similarity between the text embeddings calculated by the “*multi-qa-mpnet-base-dot-v1”* model^32,62^. The optimal number of examples for each LLM was empirically determined by assessing the model performance when different numbers of speech transcriptions were included in the input (Supplementary Fig. 2).

**Table 4:** The names and corresponding IDs in the Huggingface Hub of the three LLMs used in this study

Model	HuggingFace ID
gemma-2 (9B)	*google/gemma-2-9b*
Llama-3.1 (8B)	*meta-llama/Meta-Llama-3.1-8B*
Llama-3.1-Storm (8B)	*akjindal53244/Llama-3.1-Storm-8B*

Moreover, to test the consistency of the predictions, we used multiple paraphrases of the final prompt. The final assigned class corresponded to the most frequent one in the various predictions^33^.

Chat template (https://huggingface.co/docs/transformers/main/en/chat_templating) was used for the prompts given to the Llama-3.1-Storm (8B) model. The prompts used for each model are reported in the Supplementary Material section B.

Using a similar approach described by Portillo Wightman et al.^33^, we computed the “ON” and “OFF” confidence scores by averaging the softmax probability scores of the first token (“either ON or OFF”) provided by the models Llama-3.1 (8B) and Gemma-2 (9B).

As a final experiment, we “asked” the LLMs to explain their outcomes by defining the most influential words in the provided transcription that drove their predictions. Therefore, we replaced the last sentence of the previous prompt, “*Please provide only the class estimate without any additional text*,” with *“In addition to the class prediction, please identify and provide a list of the keywords or phrases that contributed most to the classification decision*.”

### Neuropsychiatric state score predictions using semantic search

As an initial approach to estimating the neuropsychiatric state score, we analyzed the similarities between patients’ speech transcriptions. Following the methodology described above for the semantic search, we used cosine similarity as a metric to quantify the similarity between a patient’s transcription (the query) and a corpus of transcriptions from other patients. To predict the score of a given query, we calculated a weighted average of the neuropsychiatric state scores of the top-K most similar transcriptions, using their similarity scores as weights. The results obtained using different text embeddings are reported in Supplementary Table 4.

### Neuropsychiatric state score predictions using machine learning models

We performed regression analysis to predict the neuropsychiatric state score during the two medication states. Text was mapped into embeddings using the same approach described above for the semantic search. Different regression models were used, particularly RF, ridge regression, SVM light gradient boosting machine, and extreme gradient boosting (Supplementary Table 5). We also merged the regression predictions with ensemble learning, using the bagging approach (i.e., uniformly averaging the prediction from the two best base models applied to different text embeddings)^63^. Some models predicted a neuropsychiatric state score > 60; in these cases, the values were clipped to remain consistent with the maximum possible score.

### Neuropsychiatric state score predictions using LLMs

As a third approach, we tested the capability of LLMs to perform a regression task when relevant in-context examples were provided^64^. For each LLM, we empirically investigated the optimal number of patients’ recordings (in-context examples) to include in the prompt based on the models’ performance (Supplementary Fig. 4). The K included in-context examples were the K-most similar patients’ recordings to the given query, selected using the same semantic approach explained in section above for the semantic search.

The neuropsychiatric state score was calculated as the average of nine model outputs obtained using different paraphrases of the prompt with the same in-context examples. More detailed information about the prompts used for this regression task is provided in the Supplementary Material (Section B).

### Performance evaluation

The models described above were evaluated using leave-one-out cross-validation, where two recordings from the same unseen patient served as the hold-out set.

Classification performance was assessed using accuracy, precision, recall, F1-score, and receiver operating characteristic (ROC) curve. We also provide the confidence calibration analysis of the top-performing models in Supplementary Material (Section F). Including confidence scores around a model’s prediction is especially crucial in sensitive domains like healthcare. Indeed, it has been reported that LLMs can generate incorrect answers with high confidence^65^. Therefore, calibration analysis is thus needed to assess the trustworthiness of the predictions. A model generates a well-calibrated confidence score if its predicted probabilities for a class are aligned with the actual likelihood of being correct. Calibration was tested by computing the calibration plot, the Brier score, the maximum calibration error and the expected calibration error^66,67^.

Regression performance was evaluated by computing the root mean squared error (RMSE), mean absolute error (MAE), median, interquartile range of the absolute errors, Spearman Correlation and the coefficient of determination (R^2^).

All the experiments were performed on consumer hardware equipped with a 4090 NVIDIA RTX GPU and Python version 3.11. Python’s *scikit-learn* library was used to implement ML models and evaluation metrics.

## Supplementary information

Supplementary Material

## Data Availability

The dataset generated and analyzed during the current study is not publicly available due to the sensitive nature of data but may be made available from the corresponding author on reasonable request and under the local Swiss data protection laws for research purposes only.

## Code availability

The underlying code for this study is available on GitHub and can be accessed via this link: https://github.com/matildecastelli/Neuropsychiatric_fluctuations_prediction.git.

## References

[1] Bloem, B. R., Okun, M. S. & Klein, C. Parkinson’s disease. *The Lancet* **397**, 2284–2303 (2021). 10.1016/S0140-6736(21)00218-X 33848468

[2] Weintraub, D., Matthew, M. D. & Stern, B. Psychiatric Complications in Parkinson Disease. *Am. J. Geriatr. Psychiatry* **13**, 844–851 (2005). 10.1176/appi.ajgp.13.10.844 16223962

[3] Santos García, D. et al. Non-motor symptoms burden, mood, and gait problems are the most significant factors contributing to a poor quality of life in non-demented Parkinson’s disease patients: results from the COPPADIS Study Cohort. *Parkinsonism Relat. Disord.* **66**, 151–157 (2019). 31409572 10.1016/j.parkreldis.2019.07.031

[4] Martínez-Fernández, R., Schmitt, E., Martinez-Martin, P. & Krack, P. The hidden sister of motor fluctuations in Parkinson’s disease: A review on nonmotor fluctuations. *Mov. Disord.* **31**, 1080–1094 (2016). 10.1002/mds.26731 27431515

[5] Castrioto, A., Lhommée, E., Moro, E. & Krack, P. Review mood and behavioural eff ects of subthalamic stimulation in Parkinson’s disease. *Lancet Neurol.* **13**, 287–305 (2014). 24556007 10.1016/S1474-4422(13)70294-1

[6] Pagonabarraga, J., Kulisevsky, J., Strafella, A. P. & Krack, P. Apathy in Parkinson’s disease: clinical features, neural substrates, diagnosis, and treatment. *Lancet Neurol.* **14**, 518–531 (2015). 25895932 10.1016/S1474-4422(15)00019-8

[7] Amstutz, D. et al. Management of impulse control disorders with subthalamic nucleus deep brain stimulation in Parkinson’s Disease. *CNS Neurol. Disord. Drug Targets* **19**, 611–617 (2020). 32691720 10.2174/1871527319666200720105553

[8] Debove, I. et al. Management of impulse control and related disorders in Parkinson’s Disease: an expert consensus. *Mov. Disord*. **39**, 235–248 (2024). 10.1002/mds.29700 38234035

[9] Connolly, B. S. & Lang, A. E. Pharmacological treatment of Parkinson disease: a review. *JAMA* **311**, 1670–1683 (2014). 10.1001/jama.2014.3654 24756517

[10] Rusz, J., Krack, P. & Tripoliti, E. From prodromal stages to clinical trials: the promise of digital speech biomarkers in Parkinson’s disease. *Neurosci. Biobehav. Rev.* **167**, 105922 (2024). 39424108 10.1016/j.neubiorev.2024.105922

[11] Rusz, J. et al. Quantitative assessment of motor speech abnormalities in idiopathic rapid eye movement sleep behaviour disorder. *Sleep. Med.* **19**, 141–147 (2016). 26459688 10.1016/j.sleep.2015.07.030

[12] Rusz, J., Tykalová, T., Novotný, M., Růžička, E. & Dušek, P. Distinct patterns of speech disorder in early-onset and late-onset de-novo Parkinson’s disease. *NPJ Parkinsons Dis.* **7**, 98 (2021). 34764299 10.1038/s41531-021-00243-1 PMC8585880

[13] Šubert, M. et al. Spoken language alterations can predict phenoconversion in isolated rapid eye movement sleep behavior disorder: a multicenter study. *Ann. Neurol.* **95**, 530–543 (2024). 37997483 10.1002/ana.26835

[14] Rusz, J. et al. Speech biomarkers in rapid eye movement sleep behavior disorder and Parkinson Disease. *Ann. Neurol.* **90**, 62–75 (2021). 33856074 10.1002/ana.26085 PMC8252762

[15] Pell, M. D. & Leonard, C. L. Processing emotional tone from speech in Parkinson’s disease: a role for the basal ganglia. *Cognit. Affect Behav. Neurosci.*10.3758/CABN.3.4.275 (2003). 10.3758/cabn.3.4.275 15040548

[16] Lausen, A. & Hammerschmidt, K. Emotion recognition and confidence ratings predicted by vocal stimulus type and prosodic parameters. *Humanit Soc. Sci. Commun.* **7**, 1–17 (2020).

[17] Cao, H., Beňuš, Š., Gur, R. C., Verma, R. & Nenkova, A. Prosodic cues for emotion: analysis with discrete characterization of intonation. in *Proc. International Conference on Speech Prosody* 130–134 10.21437/speechprosody.2014-14. (International Speech Communications Association, 2014). 10.21437/SpeechProsody.2014-14 PMC8042645 33855126

[18] Wan, T. M., Gunawan, T. S., Qadri, S. A. A., Kartiwi, M. & Ambikairajah, E. A comprehensive review of speech emotion recognition systems. *IEEE Access*10.1109/ACCESS.2021.3068045 (2021).

[19] Sechidis, K., Fusaroli, R., Orozco-Arroyave, J. R., Wolf, D. & Zhang, Y. P. A machine learning perspective on the emotional content of Parkinsonian speech. *Artif. Intell. Med.* **115**, 102061 (2021). 34001321 10.1016/j.artmed.2021.102061

[20] Palmirotta, C. et al. Unveiling the Diagnostic Potential of Linguistic Markers in Identifying Individuals with Parkinson’s Disease through Artificial Intelligence: A Systematic Review. *Brain Sci.*10.3390/brainsci14020137 (2024). 10.3390/brainsci14020137 PMC10886733 38391712

[21] García, A. M. et al. How language flows when movements don’t: an automated analysis of spontaneous discourse in Parkinson’s disease. *Brain Lang.* **162**, 19–28 (2016). 27501386 10.1016/j.bandl.2016.07.008

[22] Yokoi, K. et al. Analysis of spontaneous speech in Parkinson’s disease by natural language processing. *Parkinsonism Relat. Disord.* **113**, 105411 (2023). 37179151 10.1016/j.parkreldis.2023.105411

[23] Ash, S. et al. Longitudinal decline in speech production in Parkinson’s disease spectrum disorders. *Brain Lang.* **171**, 42–51 (2017). 28527315 10.1016/j.bandl.2017.05.001 PMC5512868

[24] Šubert, M. et al. Linguistic abnormalities in isolated rapid eye movement sleep behavior disorder. *Mov. Disord.* **37**, 1872–1882 (2022). 35799404 10.1002/mds.29140

[25] Cevik, F. & Kilimci, Z. H. Analysis of Parkinson’s Disease using Deep Learning and Word Embedding Models. *Acad. Perspect. Procedia* **2**, 786–797 (2019).

[26] Marras, C. et al. What Patients Say: Large-Scale Analyses of Replies to the Parkinson’s Disease Patient Report of Problems (PD-PROP). *J. Parkinsons Dis.* **13**, 757–767 (2023). 37334615 10.3233/JPD-225083 PMC10473108

[27] Agarwal, C., Tanneru, S. H. & Lakkaraju, H. Faithfulness vs. plausibility: on the (Un)Reliability of explanations from large language models. *arXiv preprint arXiv:2402.04614* (2024).

[28] Huang, S., Mamidanna, S., Jangam, S., Zhou, Y. & Gilpin, L. H. Can large language models explain themselves? A study of LLM-generated self-explanations. *arXiv preprint arXiv:2310.11207* (2023).

[29] Xie, J., Chen, A. S., Lee, Y., Mitchell, E. & Finn, C. Calibrating Language Models with Adaptive Temperature Scaling. In *Proceedings of the 2024 Conference on Empirical Methods in Natural Language Processing,* pp. 18128–18138 (2024).

[30] Schmitt, E. et al. Fluctuations in Parkinson’s disease and personalized medicine: bridging the gap with the neuropsychiatric fluctuation scale. *Front. Neurol.* **14**, 1242484 (2023). 37662035 10.3389/fneur.2023.1242484 PMC10469620

[31] Norel, R. et al. Speech-based characterization of dopamine replacement therapy in people with Parkinson’s disease. *NPJ Parkinsons Dis.* **6**, 12 (2020). 32566741 10.1038/s41531-020-0113-5 PMC7293295

[32] Zebaze, A., Sagot, B. & Bawden, R. In-Context Example Selection via Similarity Search Improves Low-Resource Machine Translation. *arXiv preprint arXiv:2408.00397* (2024).

[33] Portillo Wightman, G., DeLucia, A. & Dredze, M. *Strength in Numbers: Estimating Confidence of Large Language Models by Prompt Agreement*. In *Proceedings of the 3rd Workshop on Trustworthy Natural Language Processing (TrustNLP 2023)*, pp. 326–362, Toronto, Canada. https://github.com/JHU-CLSP/ (2023).

[34] Yang, K. et al. Towards Interpretable Mental Health Analysis with Large Language Models. In *Proceedings of the 2023 Conference on Empirical Methods in Natural Language Processing*, pp. 6056–6077 (2023).

[35] Magalhães, A. D. et al. Subthalamic stimulation has acute psychotropic effects and improves neuropsychiatric fluctuations in Parkinson’s disease. *BMJ Neurol. Open* **6**, e000524 (2024). 38196982 10.1136/bmjno-2023-000524 PMC10773312

[36] Dolev, E. L., Lutz, C. F. & Aepli, N. Does Whisper Understand Swiss German? An Automatic, Qualitative and Human Evaluation. in *Proc. Eleventh Workshop on NLP for Similar Languages, Varieties, and Dialects (VarDial 2024)* 28–40 (Association for Computational Linguistics, 2024).

[37] Sicard, C., Zürich, E., Gillioz, V. & Pyszkowski, K. Spaiche: Extending State-of-the-Art ASR Models to Swiss German Dialects. in *Proceedings of the 8th edition of the Swiss Text Analytics Conference* 76–83 (Association for Computational Linguistics, 2023).

[38] Koenecke, A., Choi, A. S. G., Mei, K. X., Schellmann, H. & Sloane, M. Careless Whisper: Speech-to-Text Hallucination Harms. in *2024 ACM Conference on Fairness, Accountability, and Transparency, FAccT 2024* 1672–1681 10.1145/3630106.3658996. (Association for Computing Machinery, Inc, 2024).

[39] Liu, C., Zhang, W., Zhao, Y., Luu, A. T. & Bing, L. Is translation all you need? A study on solving multilingual tasks with large language models. *arXiv preprint arXiv:2403.10258* (2024).

[40] Jin, R. et al. A Comprehensive Evaluation of Quantization Strategies for Large Language Models. In *Findings of the Association for Computational Linguistics: ACL 2024*, pp. 12186–12215 (2024).

[41] Han, S., Mao, H. & Dally, W. J. Deep compression: compressing deep neural networks with pruning, trained quantization and huffman coding. In *International Conference on Learning Representations* (2016).

[42] Illner, V. et al. Smartphone voice calls provide early biomarkers of Parkinsonism in rapid eye movement sleep behavior disorder. *Mov. Disord.*10.1002/mds.29921 (2024). 10.1002/mds.29921 39001636

[43] Delpont, B. et al. Psychostimulant effect of dopaminergic treatment and addictions in Parkinson’s disease. *Mov. Disord.* **32**, 1566–1573 (2017). 28737225 10.1002/mds.27101

[44] Postuma, R. B. et al. MDS clinical diagnostic criteria for Parkinson’s disease. *Mov. Disord.* **30**, 1591–1601 10.1002/mds.26424 (2015). 10.1002/mds.26424 26474316

[45] American Psychiatric Association*.* Diagnostic and Statistical Manual of Mental Disorders (DSM-5). *American Psychiatric Pub*, 5th edition. 10.1176/appi.books.9780890425596 (2013).

[46] Schade, S., Mollenhauer, B. & Trenkwalder, C. Levodopa Equivalent Dose Conversion Factors: An Updated Proposal Including Opicapone and Safinamide. *Mov. Disord. Clin. Pract*. **7**, 343–345 10.1002/mdc3.12921 (2020). 10.1002/mdc3.12921 PMC7111582 32258239

[47] Schmitt, E. et al. The neuropsychiatric fluctuations scale for Parkinson’s Disease: a pilot study. *Mov. Disord. Clin. Pract.* **5**, 265–272 (2018). 30363450 10.1002/mdc3.12607 PMC6174472

[48] Goetz, C. G. et al. Movement disorder society-sponsored revision of the unified Parkinson’s Disease Rating Scale (MDS-UPDRS): scale presentation and clinimetric testing results. *Mov. Disord.* **23**, 2129–2170 (2008). 19025984 10.1002/mds.22340

[49] Nasreddine, Z. S. et al. The montreal cognitive assessment, MoCA: a brief screening tool for mild cognitive impairment. *J. Am. Geriatr. Soc.* **53**, 695–699 (2005). 15817019 10.1111/j.1532-5415.2005.53221.x

[50] Zigmond, A. S. & Snaith, R. P. The hospital anxiety and depression scale. *Acta Psychiatr. Scand.* **67**, 361–370 (1983). 10.1111/j.1600-0447.1983.tb09716.x 6880820

[51] Starkstein, S. E. et al. Reliability, validity, and clinical correlates of apathy in Parkinson’s disease. *J. Neuropsychiatry Clin. Neurosci.* **4**, 134–139 (1992). 1627973 10.1176/jnp.4.2.134

[52] Weintraub, D. et al. Questionnaire for impulsive-compulsive disorders in Parkinson’s Disease–Rating Scale. *Mov. Disord.* **27**, 242–247 (2012). 22134954 10.1002/mds.24023 PMC3537263

[53] Rusz, J., Tykalova, T., Ramig, L. O. & Tripoliti, E. Guidelines for speech recording and acoustic analyses in dysarthrias of movement disorders. *Mov. Disord*. **36**, 803–814. 10.1002/mds.28465 (2021). 10.1002/mds.28465 33373483

[54] Radford, A. et al. Robust speech recognition via large-scale weak supervision. in *Proc. 40th International Conference on Machine Learning* (JMLR.org, 2023).

[55] Kuhn, K., Kersken, V., Reuter, B., Egger, N. & Zimmermann, G. Measuring the accuracy of automatic speech recognition solutions. *ACM Trans. Access. Comput.* **16**, 1–23 (2024).

[56] Plüss, M. et al. STT4SG-350: a speech corpus for all Swiss German Dialect Regions. in *Proc. 61st Annual Meeting of the Association for Computational Linguistics**(Volume 2: Short Papers)* vol. 2 1763–1772 (Short Papers, 2023).

[57] Etxaniz, J., Azkune, G., Soroa, A., de Lacalle, O. L. & Artetxe, M. Do multilingual language models think better in English? In *Proceedings of the 2024 Conference of the North American Chapter of the Association for Computational Linguistics: Human Language Technologies* (vol 2: Short Papers), pp. 550–564. Association for Computational Linguistics (2024).

[58] Pedregosa, F. et al. Scikit-Learn: Machine Learning in Python*.**J. Mach. Learn. Res*. **12**, http://scikit-learn.sourceforge.net (2011).

[59] Reimers, N. & Gurevych, I. Sentence-BERT: Sentence Embeddings using Siamese BERT-Networks. In *Proceedings of the 2019 Conference on Empirical Methods in Natural Language Processing and the 9th International Joint Conference on Natural Language Processing (EMNLP-IJCNLP)*, pp. 3982–3992. Association for Computational Linguistics (2019).

[60] Agbavor, F. & Liang, H. Predicting dementia from spontaneous speech using large language models. *PLOS Digital Health* **1**, e0000168 (2022). 36812634 10.1371/journal.pdig.0000168 PMC9931366

[61] Vu, H., Abdurahman, S., Bhatia, S. & Ungar, L. Predicting Responses to Psychological Questionnaires from Participants’ Social Media Posts and Question Text Embeddings. In *Findings of the Association for Computational Linguistics: EMNLP 2020*. 10.18653/v1/2020.findings-emnlp.137 (2020).

[62] Mathur, Y. et al. SummQA at MEDIQA-Chat 2023: In-Context Learning with GPT-4 for Medical Summarization. In *Proceedings of the 5th Clinical Natural Language Processing Workshop*, pp. 490–502, Association for Computational Linguistics (2023).

[63] Naderalvojoud, B. & Hernandez-Boussard, T. Improving machine learning with ensemble learning on observational healthcare data. *AMIA Annual Symposium Proceedings,* 521–529 (2024). PMC10785929 38222353

[64] Vacareanu, R., Negru, V.-A., Suciu, V. & Surdeanu, M. From words to numbers: your large language model is secretly a capable regressor when given in-context examples. arXiv preprint arXiv:2404.07544 (2024).

[65] Jiang, Z., Araki, J., Ding, H. & Neubig, G. How Can We Know When Language Models Know? On the Calibration of Language Models for Question Answering. *Trans. Assoc. Comput. Linguist* 962–977 10.1162/tacl (2021).

[66] Pereira, T., Cardoso, S., Guerreiro, M., Mendonça, A. & Madeira, S. C. Targeting the uncertainty of predictions at patient-level using an ensemble of classifiers coupled with calibration methods, Venn-ABERS, and Conformal Predictors: A case study in AD. *J. Biomed. Inf.* **101**, 103350 (2020). 10.1016/j.jbi.2019.103350 31816401

[67] Guo, C., Pleiss, G., Sun, Y. & Weinberger, K. Q. On calibration of modern neural networks. in *Proc.Machine Learning Research* 1321–1330 (PMLR, 2017).

