# Supplementary material for: Detecting neuropsychiatric fluctuations in Parkinson’s Disease using patients’ own words: the potential of large language models
Source: NPJ Parkinsons Dis. 2025 Apr 18;11:79. doi: 10.1038/s41531-025-00939-8 (PMC12008272; doi:10.1038/s41531-025-00939-8)
Supplement: Supplementary file 1 — Supplementary Material [file 41531_2025_939_MOESM1_ESM.pdf]

Supplementary Material

Section A: Selecting the optimal number of retrieved NFS items

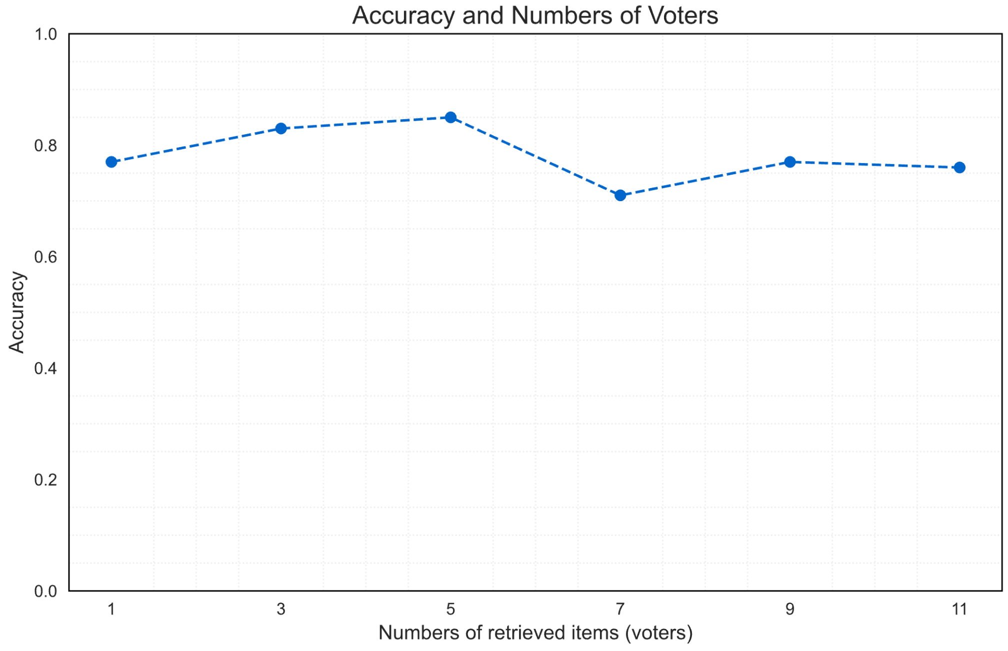

Supplementary Fig. 1: Accuracy of the Semantic Search approach described in section 2.5.1 in function of the number of voters. The accuracies are quite similar, with the best accuracy obtained considering the 5 most similar items as the voters.

Section B: Prompts

We tested various prompts and prompt strategies before finalizing the versions reported here. Initially, we began with a straightforward instruction to classify the text as either positive or negative neuropsychiatric condition. We then included demonstrations (transcriptions), modified the main classification instructions to classify as ON or OFF, and incorporated context by using the most common NFS items described in the original NFS validation study.

The prompt for ON-OFF classification with LLMs model:

"*You will be provided with transcription of speech from people with Parkinson's Disease. Please assign the class ON or OFF to each text. Patients in the OFF condition may experience tiredness, lack of energy, inability to relax, trembling legs, and feel lethargic and low. In the ON condition, they usually feel full of energy, sure of themselves, talkative, and a sense of well-being. Please provide only the class estimate, without any additional text.*

Transcription 1: …
Class: ON

Transcription 2: …
Class: ON

Transcription 3: …

Class: ON

Transcription 4: …
Class: OFF

Transcription: {new transcription}
Class:"

The regression prompt for Llama-3.1-8B and Gemma2-9b models:
"*You will be provided with transcription of speech from people with Parkinson's Disease. Please assign to each text a neuropsychiatric score between 0 and 60. The score is lower if the patients experience tiredness, lack of energy, inability to relax, trembling legs, and feel lethargic and low. The score is higher if the patient feels full of energy, sure of themselves, talkative, and a sense of well-being."*

Trascription 1: …
Score: 29

Trascription 2:
Score: 17

Trascription 3:
Score 4

Trascription: {new transcription}
Score:"

Prompt with chat template for Llama 3.1-Storm-8B model:
messages = [
{"role": "system", "content": "You are a helpful medical assistant. You will be provided with a transcription from a patient with Parkinson's Disease.},
{"role": "user," "content": "Please assign the class ON or OFF to each text. Patients in the OFF condition may experience tiredness, lack of energy, inability to relax, trembling legs, and feel lethargic and low. In the ON condition, they usually feel full of energy, sure of themselves, talkative, and a sense of well-being. Please provide only the class estimate, without any additional text.},
{"role": "user", "content": "I feel tired. Tired and heavy legs...},
{"role": "assistant", "content": "OFF "},
{"role": "user", "content": "At the moment I'm a bit blocked…}
{"role": "assistant", "content": "OFF "},
{"role": "user", "content": new_transcription},
]

Examples of prompts Paraphrases
The paraphrases were generated using the GPT-4o model and then manually adjusted.

“You will be provided with speech transcription from people with Parkinson’s Disease. Please assign the class ON or OFF to each text. Examples of OFF features are related to tiredness, lacking energy, inability to relax, and trembling legs. ON features: well-being, being full of energy, being confident, and being talkative.”

“Classify each provided speech transcription from Parkinson’s patients as either ON or OFF. Note: OFF includes fatigue, low energy, difficulty relaxing, and trembling legs; ON includes well-being, high energy, confidence, and talkativeness.”

“Assign the label ON or OFF to the following text transcriptions from individuals with Parkinson’s Disease. OFF characteristics: tiredness, low energy, inability to relax, trembling legs; ON characteristics: well-being, high energy, confidence, talkativeness.”

“Classify the given speech transcriptions from individuals with Parkinson’s as either ON or OFF. OFF traits include tiredness, low energy, inability to relax, trembling legs; ON traits include well-being, high energy, confidence, and talkativeness.”

Section C: Additional Methods ON/OFF Medication State Classification

Supplementary Table 1 Performance of semantic search approach using different text embeddings and corpus.

Corpus	Text Embeddings Model	Accuracy	Precision	Recall	F1 macro
**NFS items**	CountVectorizer	0.700	0.700	0.670	0.680
**NFS items**	Tfidfvectorizer	0.670	0.670	0.669	0.667
**NFS items**	multi-qa-mpnet-base-dot-v1	**0.848**	**0.857**	**0.848**	**0.848**
**NFS items**	stella_en_1.5B_v5	0.561	0.602	0.561	0.511
**NFS items**	gte-Qwen2-1.5B-instruct	0.712	0.751	0.712	0.701
**Patients’ recordings**	CountVectorizer	0.801	0.822	0.801	0.801
**Patients’ recordings**	TfidfVectorizer	0.800	0.800	0.809	0.780
**Patients’ recordings**	multi-qa-mpnet-base-dot-v1	0.801	0.822	0.801	0.801
**Patients’ Recordings**	stella_en_1.5B_v5	0.621	0.665	0.621	0.594
**Patients’ Recordings**	gte-Qwen2-1.5B-instruct	0.727	0.727	0.727	0.727

The first two rows report the performance metrics obtained when the NFS items were used as the corpus. The last row represents the metrics achieved when the corpus was composed of the patients' recordings, excluding the query recording.

Supplementary Table 2. Results of machine learning approach using different text embedding and machine learning classifiers.

Text Embeddings	Model	Accuracy	Precision	Recall	F1 macro
CountVectorizer (Original Dimensions: 3813)	RF	0.807	0.828	0.807	0.803
	SVM	0.833	0.836	0.833	0.829
CountVectorizer (Dim after dimensionality reduction: 60)	RF	0.812	0.823	0.818	0.818
	SVM	0.833	*0.836*	0.833	0.833
	GaussianNB	0.682	0.686	0.682	0.680
TfidfVectorizer (Dim after dimensionality reduction: 60)	RF	0.788	0.788	0.788	0.788
	SVM	*0.848*	*0.854*	*0.848*	*0.848*
	GaussianNB	0.530	0.530	0.530	0.530
multi-qa-mpnet-base-dot-v1 (Dim: 768; PCA dim: 40)	RF	0.818	0.823	0.818	0.818
	SVM	*0.909*	*0.911*	*0.909*	*0.909*
	Gaussian NB	0.758	0.759	0.758	0.757
stella_en_1.5B_v5 (Dim: 1024, PCA dim: 40	RF	0.894	0.894	0.894	0.894
	SVM	*0.909*	*0.911*	*0.909*	*0.911*
	GaussianNB	0.621	0.621	0.621	0.621
gte-Qwen2-1.5B-instruct (Dim: 1536; PCA dim: 45)	RF	0.924	0.925	0.924	0.924
	SVM	0.924	0.925	0.924	0.924
	Gaussian NB	0.758	0.782	0.758	0.753

Only the best models resulting from Grid Search are displayed. The first column contains the HuggingFace ID of the model, and the resulting vector dimension before and after the application of the PCA is shown. Truncated singular value decomposition method was used to reduce the dimensionality of CountVectorizer and TfidfVectorizer.
RF = Random Forest, SVM = Support Vector Machine.

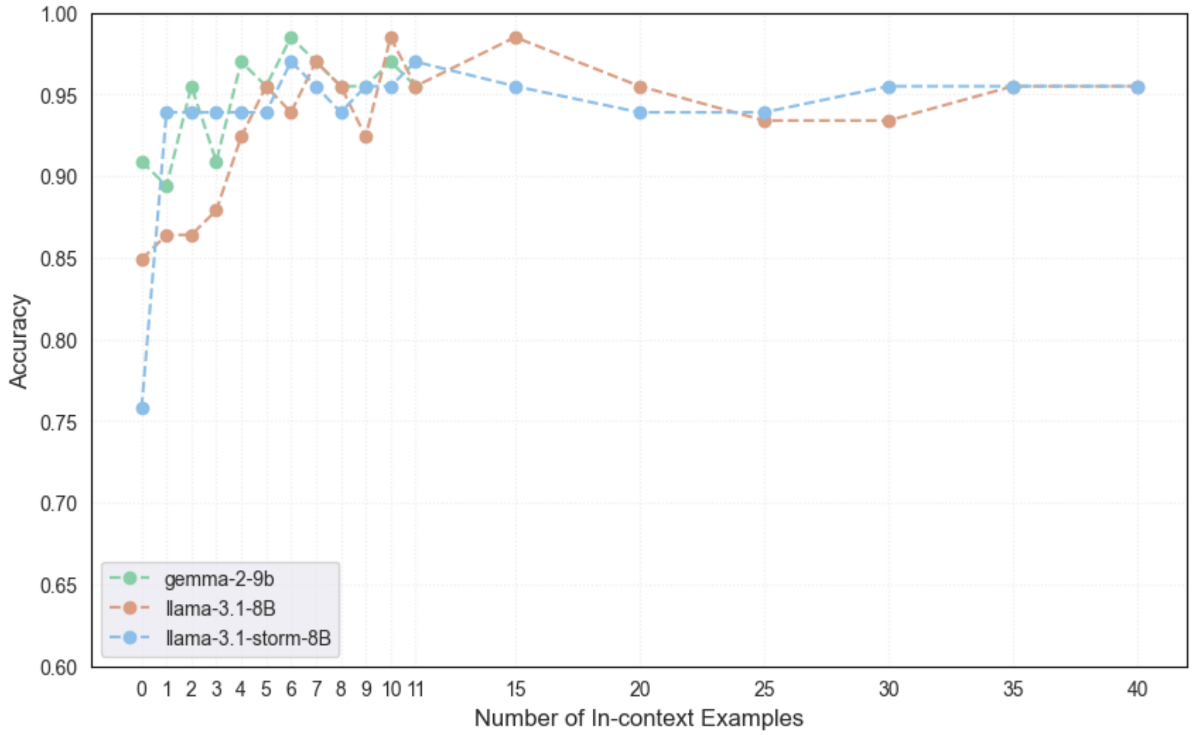

Supplementary Fig. 2: LLMs Accuracy by the number of in-context examples. The accuracy changed based on the number of similar patient recordings in the prompt. Gemma-2-9b has a context length of 8192; thus, up to 11 transcriptions were added to the prompt. The llama models, instead, support a context length of 128K tokens. After ten in-context examples, the llama models converged to an accuracy value of around 0.95.

Model	Accuracy	Precision	Recall	F1 macro
Llama-3.1-Storm-8B (0-shot)	0.758	0.979	0.758	0.854
Llama-3.1-Storm-8B (few-shot)	0.969	0.971	0.970	0.961
Llama-3.1-8B (0-shot)	0.849	0.860	0.849	0.849
Llama-3.1-8B (few-shot)	**0.985**	**0.985**	**0.985**	**0.985**
Gemma-2-9b (0-shot)	0.909	0.909	0.909	0.909
Gemma-2-9b (few-shot)	**0.985**	**0.985**	**0.985**	**0.985**

Supplementary Table 3. Performance of different LLMs including zero and few examples in the prompts. All the models achieved an accuracy greater than 0.95 when few examples were included in the prompt, specifically 6 examples for the gemma-2-9b, 10 for llama-3.1-8B and 6 for the llama-3.1-storm.

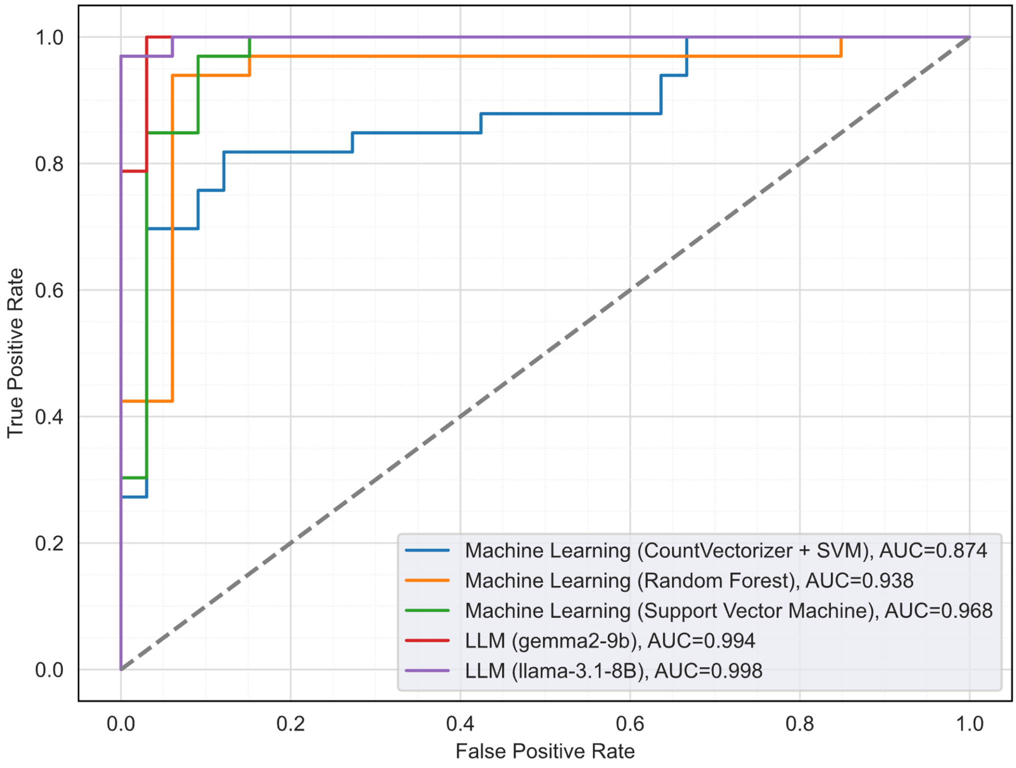

Supplementary Fig. 3: AUC-ROC curve of the baseline model (CountVectorizer with dimensionality reduction + Support Vector Machine) and top-performing machine learning and LLMs approaches.

Section D: Neuropsychiatric State Score Prediction

Corpus	Text Embeddings	RMSE	MAE ± STD (AE Median, Q1-Q3)	Spearman Correlation	R^2
Patients' Recordings	CountVectorizer	15.5	12.5 ± 9.3 (11.1,4.9-18.1)	0.56	0.30
Patients' Recordings	TfidfVectorizer	16.5	13.5 ± 9.3 (11.6,7.6-18.4)	0.46	0.22
Patients' Recordings	multi-qa-mpnet-base-dot-v1	14.0	11.7 ± 7.9 (11.0,5.25-17)	0.64	0.43
Patients' Recordings	stella_en_1.5B_v5	18.8	15.4 ± 10.9 (12.5,8.3-21)	0.29	-0.02
Patients' Recordings	gte-Qwen2-1.5B-instruct	17.3	13.7 ± 10.6 (12.3,3.38-22.9)	0.49	0.22

Supplementary Table 4 Semantic Search approach results for neuropsychiatric state score prediction. RMSE and MAE with Standard deviation are provided. The median and interquartile range of the absolute errors (AE) are reported below the MAE.

Text Embeddings	Model	RMSE	MAE ± STD (AE Median, Q1-Q3)	Spearman Correlation	R^2
**CountVectorizer**<br><br>**Dimensions: 60**	Ridge	15.1	12.6 ± 8.4 (10.7,6.6-17.5)	0.63	0.34
	RF	16.4	14.2 ± 8.2 (13.3,8.1-19.6)	0.53	0.23
	SVM	13.9	11.3 ± 8.3 (9.6,4.1-15.8)	0.67	0.44
	XGBoost	15.1	13.2 ± 9.9 (11.7,6.1-16.5)	0.57	0.34
	LightGBM	16.5	14.5 ± 8.0 (14.7,7.8-19.9)	0.38	0.21
**TfidfVectorizer**<br><br>**Dimensions: 60**	Ridge	17.4	15.5 ± 7.9 (15.6,9.3-21.5)	0.42	0.13
	RF	17.9	16.2 ± 7.6 (16.3,10.3-21.9)	0.38	0.07
	SVM	17.4	15.2 ± 8.6 (15.9,8.8-21.2)	0.32	0.12
	XGBoost	16.4	13.3 ± 9.7 (12.3,5.5-20.2)	0.46	0.22
	LightGBM	15.2	12.1 ± 9.2 (9.7,5.55-17.4)	0.57	0.33
**multi-qa-mpnet-base-dot-v1**<br><br>PCA dimensions: 41	Ridge	17.4	14.1 ± 10.2 (11.8, 4.6-21.3)	0.55	0.13
	RF	13.9	11.0 ± 8.5 (10.2, 4.6-14.9)	0.65	0.44
	SVM	14.0	11.8 ± 7.7 (9.2, 5.8-17.2)	0.67	0.43
	XGBoost	14.9	11.5 ± 9.5 (9.2,3.9-17.2)	0.59	0.36
	LightGBM	15.4	12.0 ± 9.6 (10.3,3.6-17.6)	0.57	0.32
stella_en_1.5B_v5<br><br>PCA dimensions: 40	Ridge Regression	13.5	10.4 ± 8.7 (7.0, 3.5-16.0)	0.69	0.47
	RF	14.0	10.9 ± 8.9	0.67	0.43

			(8.8, 3.9-14.8)		
	SVM	12.6	10.3 ± 7.4 (8.7, 3.1-16.1)	0.75	0.54
	XGBoost	13.9	10.7 ± 9.0 (9.5, 3.5-14.2)	0.64	0.44
	LightGBM	14.6	12.4 ± 8.1 (10.2, 6.3-17.1)	0.65	0.39
gte-Qwen2-1.5B-instruct	Ridge Regression	16.6	13.7 **±** 9.5 (13.6, 6.7-17.6)	0.59	0.21
PCA dimensions:45	**RF**	**11.2**	**8.7 ± 7.1 (6.2, 3.1-**13.4**)**	**0.76**	**0.64**
	SVM	12.6	10.1 ± 7.5 (8.6,4.3-14.8)	0.72	0.54
	XGBoost	12.1	9.6 ± 7.1 (8.8, 3.4-14.2)	0.71	0.57
	LightGBM	12.6	9.8 ± 7.1 (7.6, 4.4-14.1)	0.73	0.58
gte-Qwen2-1.5B-instruct + stella_en_1.5B_v5	Ensemble: Random Forests + LightGBM	11.9	9.5 ± 7.1 (7.6, 3.3-15.5)	0.74	0.57

Supplementary Table 5 Machine Learning Approach results for neuropsychiatric state score prediction obtained by Leave-one-out-cross-validation. RMSE and MAE with Standard deviation are provided. The median and interquartile range of the absolute errors are reported below the MAE.

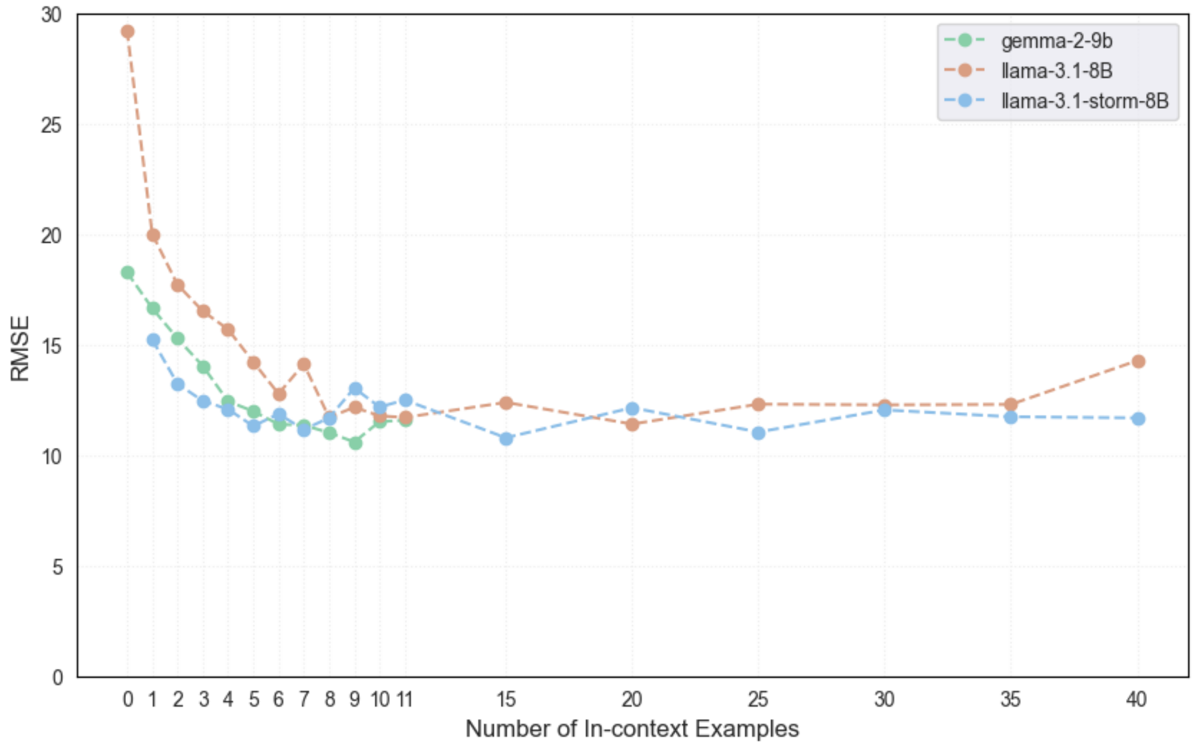

Supplementary Fig. 4: LLMs performance in estimating the neuropsychiatric state score based on the number of in-context examples. Of note, the llama-3.1-storm-8B gave coherent responses only when examples were included in the prompt. Thus, the plot does not report the Root Mean Squared Error (RMSE) in the 0-shot setting. Gemma-2-9b has a context length of 8192; thus, up to 11 transcriptions were added to the prompt. The llama models, instead, support a context length of 128K tokens. After 10 in-context examples, the models converged to an RMSE value of approximately 11.

**Model**	**RMSE**	**MAE ± STD (AE Median, Q1-Q3)**	**Spearman Correlation**	**R^2**
Gemma-2-9b (few-shot)	**10.6**	**8.1 ± 6.9 (6.0, 3.0-10.4)**	**0.81**	**0.68**
Llama-3.1-Storm-8B (few-shot)	11.2	8.4 ± 7.4 (6.7, 3.0-11.8)	0.80	0.64
Llama-3.1-8B (few-shot)	11.4	8.4 ± 7.8 (6.3, 3.1-11.2)	0.80	0.62

Supplementary Table 6 LLM Approach results for neuropsychiatric state score prediction. RMSE and MAE with Standard deviation are provided. The median and interquartile range of the absolute errors are reported below the MAE. All the models achieved lower errors when few examples were included in the prompt. In this table are reported the best results obtained by the 3 LLMs, specifically, the “gemma-2-9b” in the 9-shot setting, “llama-3.1-8B” in the 20-shot setting, and “llama-3.1-storm” in the 6-shot setting.
RMSE = Root Mean Squared Error, MAE = Mean Absolute Error, AE = Absolute Error, Q1 = First quartile, Q3 = Third quartile.

Section E: Random selection of In-Context examples and top-k semantically similar

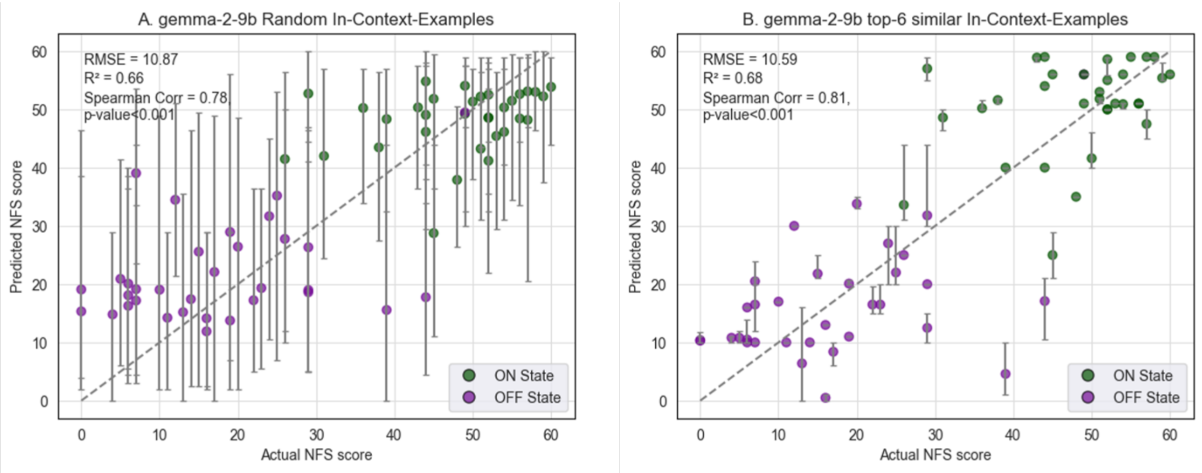

Supplementary Fig. 5: Scatter plots of predicted vs actual neuropsychiatric state score. The LLM's predictions were extremely sensitive to the in-context examples provided in the input. The grey continuous error bars represent the range between the 5th and 95th percentile of nine predictions. The results were obtained in the 9-shot scenario. **a)** Gemma-2-9b average predictions with error bars when nine in-context examples are randomly selected**.** The random selection of transcriptions led to highly variable neuropsychiatric state score estimates. **b)** Gemma-2-9b average predictions with error bars when different instruction paraphrases are used, and the nine most similar examples are selected. Paraphrasing the prompt and keeping the same, most similar examples to the query improved the correlation and decreased the errors.

Section F: Calibration Analysis

Model	Brier Score	ECE (bins=10)	MCE
**LLM (Llama-3.1-8B)**	0.43	**0.08**	0.42
**LLM (Gemma2-9B)**	0.39	0.13	0.43
**Random Forest** (Embedding model: Alibaba/gte-Qwen2-1.5B-instruct)	**0.27**	0.30	0.45
**Support Vector Machine** (Embedding model: Alibaba/gte-Qwen2-1.5B-instruct)	0.39	0.09	**0.37**

Supplementary Table 7 Calibration of the best models, including the Brier Score, Expected Calibration Error and Maximum Calibration error. For all these metrics, the lower, the better.

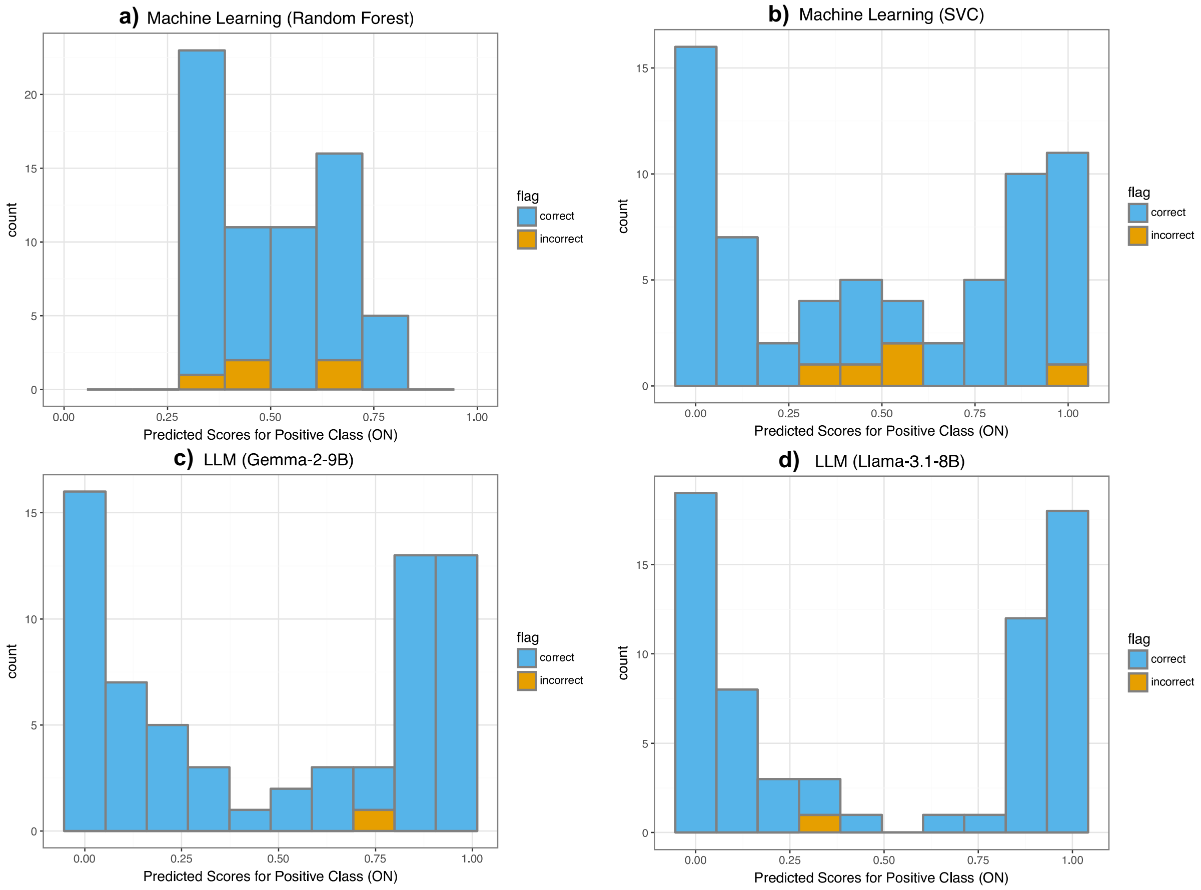

Supplementary Fig. 6: Qualitative representation of predicted scores for the positive class (ON), computed by the best models.

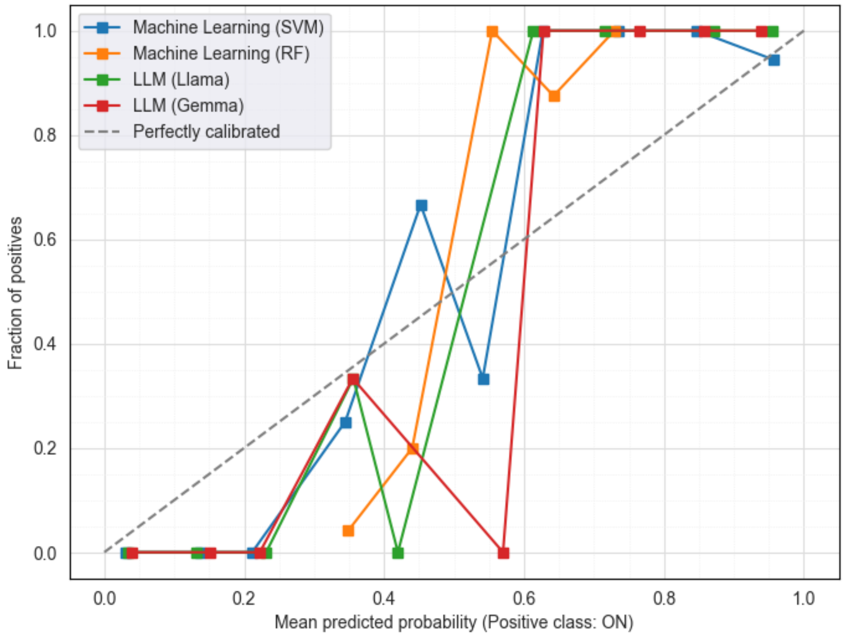

Supplementary Fig. 7: Calibration plots of the best classification models.

Section G: Replication of recordings in the OFF condition

Seven patients were recorded two times in the OFF medication condition.

These seven recordings in the OFF medication condition were treated as the test set for the medication classification and NFS score prediction tasks.

Medication State Classification

Model	Accuracy
**Machine Learning** (SVM + Alibaba-NLP/gte-Qwen2-1.5B-instruct embeddings)<br><br>**Machine Learning** (RF+ Alibaba-NLP/gte-Qwen2-1.5B-instruct)	0.86 (1 error)
**LLM** (gemma2-9b)	1.0 (100%)

Supplementary Table 8. Accuracy of the best models on a test set of 7 patients recorded a second time in OFF.

Neuropsychiatric state prediction

Machine Learning (Random Forest + Alibaba-NLP/gte-Qwen2-1.5B-instruct embeddings)

Mean absolute error OFF1 = 7.3
Mean absolute error OFF2 = 7.5

ID	Acutal NFS	NFS prediction OFF1	NFS prediction OFF2
P1	0	17.1	15.9
P3	7	23.4	16.7
P23	29	22.7	18.9

P15	22	19.5	17.7
P27	24	21.5	28.7
P32	23	16.9	19.2
P10	19	17.7	21.7

Supplementary Table 9. Actual Neuropsychiatric State score vs predicted Neuropsychiatric score by random forest.

LLM (Gemma-2-9B)

Mean absolute error OFF1 = 5.8
Mean absolute error OFF2 = 8.4

ID	Acutual NFS	NFS prediction OFF1	NFS prediction OFF2
P1	0	10.7	10.4
P3	7	20.5	27.2
P23	29	31.8	29.2
P15	22	16.5	11
P27	24	24	27
P32	23	16.5	10.2
P10	19	20.7	20.1

Supplementary Table 10. Actual Neuropsychiatric State score vs predicted Neuropsychiatric score by the LLM.

Section H: Models Hyperparameters

Model	Hyperparameters Ranges
**Random Forest Classifier** (random_state = 0)	"n_estimators": [100, 200, 500], "max_features": ["sqrt", "log2"], "max_depth": [3,4,5,6,7,8], "criterion": ["gini", "entropy"]
**Random Forest Regressor** (random_state = 0)	"RandomForestRegressor": { "n_estimators": [100, 200, 500,600], "max_features": ["sqrt", "log2",1.0], "max_depth": [4,5,6,7,8], "criterion": ["squared_error", "absolute_error"]
**SVM Classifier**	"C": [0.001, 0.01, 0.1, 1, 10, 100], "gamma": ["auto","scale",0.1, 1, 10], "degree": [1, 2, 3, 4], "kernel": ["linear", "poly", "rbf","sigmoid"]
**SVM Regressor**	"C": [0.01, 0.1, 1, 10, 100], "gamma": ["auto","scale",0.1, 1, 10], "degree": [1, 2, 3, 4], "kernel": ["linear", "poly", "rbf","sigmoid"]
**Gaussian NB**	"var_smoothing": [1e-10, 1e-9, 1e-8,1e-5]
**Ridge Regression**	"alpha": [1e-15,1e-10,1e-8,1e-3,1e-2,1,5,10,20,30,35,40,45,50,55,100]
**XGBoost Regression**	"n_estimators": [50, 100, 200, 500], "learning_rate": [0.01, 0.1, 0.2], "max_depth": [3, 4, 5, 7],

	"subsample": [0.8, 1.0],<br>"colsample_bytree": [0.8, 1.0]
**LightGBM**	"num_leaves": [15, 31, 63],<br>"max_depth": [3, 6, 9],<br>"learning_rate": [0.01, 0.1, 0.2],<br>"n_estimators": [100, 200, 500],<br>"subsample": [0.8, 1.0],<br>"colsample_bytree": [0.8, 1.0],<br>"lambda_l1": [0, 1, 5],<br>"lambda_l2": [0, 1, 5]

Supplementary Table 11. Hyperparameter ranges for GridSearchCV hyperparameter tuning of machine learning models.

Embedding Model	ML Model	Hyperparameters
CountVectorizer	RF	{'criterion': 'gini', 'max_depth': 4, 'max_features': 'sqrt', 'n_estimators': 200}
	SVM	{'C': 0.001, 'gamma': 0.1, 'kernel': 'sigmoid'}
CountVectorizer (with dimensionality reduction)	RF	{'criterion': 'entropy', 'max_depth': 6, 'max_features': 'sqrt', 'n_estimators': 100}
	SVM	{'C': 10, 'gamma': 'scale', 'kernel': 'rbf'}
	GaussianNB	{'var_smoothing': 1e-10}
TfidfVectorizer	RF	{'criterion': 'gini', 'max_depth': 7, 'max_features': 'sqrt', 'n_estimators': 100}
	SVM	{'C': 0.1, ‘degree’:2,'gamma': 10, 'kernel': 'poly'}
	GaussianNB	{'var_smoothing': 1e-10}
multi-qa-mpnet-base-dot-v1	RF	{'criterion': 'entropy', 'max_depth': 4, 'max_features': 'sqrt', 'n_estimators': 100}
	SVM	{'C': 10, 'gamma': 'auto', 'kernel': 'sigmoid'}
	GaussianNB	{'var_smoothing': 1e-10}
stella_en_1.5B_v5	RF	{'criterion': 'entropy', 'max_depth': 4, 'max_features': 'sqrt', 'n_estimators': 200}
	SVM	{'C': 1, 'degree': 1, 'gamma': 'scale', 'kernel': 'poly'}
	GaussianNB	{'var_smoothing': 1e-10}

gte-Qwen2-1.5B-instruct	RF	{'criterion': 'gini', 'max_depth': 4, 'max_features': 1.0, 'n_estimators': 500}
	SVM	{'C': 0.001, 'degree': 1, 'gamma': 10, 'kernel': 'poly'}
	GaussianNB	{'var_smoothing': 1e-10}

Supplementary Table 12. Best Hyperparameter combinations for the classification task.

Embedding Model	ML Model	Hyperparameters
CountVectorizer	Ridge	{'alpha': 10}
	RF	{'criterion': 'absolute_error', 'max_depth': 4, 'max_features': 'sqrt', 'n_estimators': 200}
	SVM	{'C': 1, 'degree': 1, 'gamma': 10, 'kernel': 'sigmoid'}
	XGBoost	{'colsample_bytree': 0.8, 'learning_rate': 0.01, 'max_depth': 3, 'n_estimators': 500, 'subsample': 0.8}
TfidfVectorizer	Ridge	{'alpha': 1}
	RF	{'criterion': 'absolute_error', 'max_depth': 6, 'max_features': 'log2', 'n_estimators': 100}
	SVM	{'C': 100, 'gamma': 0.1, 'kernel': 'rbf'}
	XGBoost	{'colsample_bytree': 0.8, 'learning_rate': 0.01, 'max_depth': 3, 'n_estimators': 500, 'subsample': 0.8}
multi-qa-mpnet-base-dot-v1	Ridge	{'alpha': 100}
	RF	{'criterion': 'squared_error', 'max_depth': 7, 'max_features': 'sqrt', 'n_estimators': 100}
	SVM	{'C': 100, 'degree': 1, 'gamma': 'scale', 'kernel': 'rbf'}
	XGBoost	{'colsample_bytree': 0.8, 'learning_rate': 0.2, 'max_depth': 3, 'n_estimators': 500, 'subsample': 1.0}
stella_en_1.5B_v5	Ridge	{'alpha': 100}
	RF	

		{'criterion': 'squared_error', 'max_depth': 8, 'max_features': 1.0, 'n_estimators': 200}
	SVM	{'C': 10, 'degree': 1, 'gamma': 'scale', 'kernel': 'poly'}
	XGBoost	{'colsample_bytree': 1.0, 'learning_rate': 0.01, 'max_depth': 7, 'n_estimators': 500, 'subsample': 0.8}
Alibaba	Ridge	{'alpha': 100}
	RF	{'criterion': 'absolute_error', 'max_depth': 4, 'max_features': 1.0, 'n_estimators': 500}
	SVM	{'C': 10, 'gamma': 'scale', 'kernel': 'poly'}
	XGBoost	{'colsample_bytree': 1.0, 'learning_rate': 0.01, 'max_depth': 4, 'n_estimators': 500, 'subsample': 1.0}
	LightGBM	{'colsample_bytree': 1.0, 'lambda_l1': 5, 'lambda_l2': 1, 'learning_rate': 0.1, 'max_depth': 3, 'n_estimators': 500, 'num_leaves': 15, 'subsample': 0.8}

Supplementary Table 13. Best Hyperparameter combinations for the regression task.

Parameters of generate method	Value	Short Explanation
Do_sample	False	Decoding greedy Approach
max_new_tokens	4	Reduce the length of the generated response
return_dict_in_generate	True	To return the output transition scores, i.e. the token log-probabilities
output_scores	True	“”

Supplementary Table 14. Parameters LLMs set during inference time.
